# Next-Generation Sequencing-Based Quantitative Detection of Hepatitis B Virus Pre-S Mutants in Plasma Predicts Hepatocellular Carcinoma Recurrence

**DOI:** 10.3390/v12080796

**Published:** 2020-07-24

**Authors:** Chiao-Fang Teng, Tsai-Chung Li, Hsi-Yuan Huang, Jia-Hui Lin, Wen-Shu Chen, Woei-Cherng Shyu, Han-Chieh Wu, Cheng-Yuan Peng, Ih-Jen Su, Long-Bin Jeng

**Affiliations:** 1 Graduate Institute of Biomedical Sciences, China Medical University, No.91, Hsueh-Shih Rd., Northern Dist., Taichung City 404, Taiwan; estherlin292@gmail.com (J.-H.L.); shyu9423@gmail.com (W.-C.S.); 2 Organ Transplantation Center, China Medical University Hospital, No.2, Yude Rd., Northern Dist., Taichung City 404, Taiwan; d00443003@gmail.com; 3 Research Center for Cancer Biology, China Medical University, Taichung City 404, Taiwan; 4 Department of Public Health, College of Public Health, China Medical University, Taichung City 404, Taiwan; tcli@mail.cmu.edu.tw; 5 Department of Healthcare Administration, College of Medical and Health Science, Asia University, Taichung City 413, Taiwan; 6 Department of Laboratory Medicine, China Medical University Hospital, Taichung City 404, Taiwan; cn0312@gmail.com; 7 Department of Occupational Therapy, Asia University, Taichung City 413, Taiwan; 8 Department of Neurology, China Medical University Hospital, Taichung City 404, Taiwan; 9 Translational Medicine Research Center, China Medical University Hospital, Taichung City 404, Taiwan; 10 National Institute of Infectious Diseases and Vaccinology, National Health Research Institutes, Zhunan 350, Taiwan; hanjie@nhri.org.tw; 11 Division of Hepatogastroenterology, Department of Internal Medicine, China Medical University Hospital, Taichung City 404, Taiwan; cypeng@mail.cmuh.org.tw; 12 School of Medicine, China Medical University, Taichung City 404, Taiwan; 13 Department of Biotechnology, Southern Taiwan University of Science and Technology, No.1, Nantai St., Yongkang Dist., Tainan City 710, Taiwan

**Keywords:** hepatocellular carcinoma, hepatitis B virus, pre-S mutants, recurrence, next-generation sequencing

## Abstract

Hepatocellular carcinoma (HCC) is among the most common and lethal human cancers worldwide. Despite curative resection, high recurrence of HCC remains a big threat, leading to poor patient outcomes. Hepatitis B virus (HBV) pre-S mutants, which harbor deletions over pre-S1 and pre-S2 gene segments of large surface proteins, have been implicated in HCC recurrence. Therefore, a reliable approach for detection of pre-S mutants is urgently needed for predicting HCC recurrence to improve patient survival. In this study, we used a next-generation sequencing (NGS)-based platform for quantitative detection of pre-S mutants in the plasma of HBV-related HCC patients and evaluated their prognostic values in HCC recurrence. We demonstrated that the presence of deletions spanning the pre-S2 gene segment and the high percentage of pre-S2 plus pre-S1 + pre-S2 deletions, either alone or in combination, was significantly and independently associated with poor recurrence-free survival and had greater prognostic performance than other clinicopathological and viral factors in predicting HCC recurrence. Our data suggest that the NGS-based quantitative detection of pre-S mutants in plasma represents a promising approach for identifying patients at high risk for HBV-related HCC recurrence after surgical resection in a noninvasive manner.

## 1. Introduction

Hepatocellular carcinoma (HCC) is the sixth most frequent human cancer and the second leading cause of cancer-related death worldwide, responsible for around 740,000 deaths in 2012 [1,2,3]. Many therapeutic strategies have been developed for the treatment of HCC, among which surgical resection is regarded as one of the best therapeutic options for HCC patients [4,5,6,7]. However, the recurrence rate of HCC after curative surgical resection is up to 80% within five years, leading to a poor patient survival rate as low as 30% within five years [8,9,10]. Therefore, identification of effective and reliable biomarkers for the prediction of HCC recurrence after surgical resection is urgently needed, allowing for earlier and better prevention and management of the recurrent HCC to improve patient survival.

Chronic infection with hepatitis B virus (HBV) is a major risk factor for HCC development, accounting for over 50% of total HCC cases in developing countries [11,12]. Over the past decade, we have identified ground glass hepatocytes (GGHs) in liver tissues as a histological marker of chronic HBV infection as well as the preneoplastic lesions of HBV-associated HCC [13]. Two different types of GGHs (designated types I and II) consistently express distinct inclusion-like and marginal patterns of pre-S mutant proteins (pre-S1 and pre-S2 mutants), which contain deletion mutations over the pre-S1 and pre-S2 gene segments of HBV large surface proteins, respectively [14,15]. We have well demonstrated that both types of pre-S mutants are accumulated in the endoplasmic reticulum (ER) of hepatocytes in patients with chronic HBV infections and can induce multiple ER stress-dependent and -independent signal pathways, resulting in genomic instability and growth advantages of hepatocytes and eventually HCC formation [13,16,17]. The prevalence of pre-S mutants in liver tissues and serum/plasma samples of chronic HBV carriers is approximately 37%, and is gradually increased with disease progression, and eventually accounts for up to 60% in HCC patients [13,18]. The presence of pre-S mutants in chronic HBV carriers confers a 5-fold higher risk for HCC development [18,19,20]. Moreover, HBV-related HCC patients carrying pre-S mutants have a significantly greater risk of HCC recurrence after surgical resection, even after receiving postoperative antiviral therapy [21,22,23,24]. As a result, the presence of pre-S mutants represents an important high-risk biomarker for HCC development in chronic HBV carriers as well as HCC recurrence in HBV-related HCC patients following surgical resection.

Three major approaches have been successfully applied to detect pre-S mutants in liver tissues or serum/plasma samples for identifying patients with high risk of HCC development and recurrence, one based on the immunohistochemistry staining of HBV surface antigens (HBsAg; surface proteins) for GGHs visualization [15,22], one utilizing polymerase chain reaction (PCR) amplification of pre-S gene for DNA sequencing [19,20,21], and the usage of the Pre-S Gene Chip for hybridization with pre-S gene PCR products [18,24]. However, these approaches provide only qualitative and semi-quantitative detection results and remain in need of improvement. Therefore, we have recently developed a next-generation sequencing (NGS)-based platform for quantitative detection of pre-S mutants in plasma samples of HBV-related HCC patients with better sensitivity and accuracy [25,26]. In this study, we further evaluated this NGS-based quantitative detection of pre-S mutants as a powerful approach to predict HCC recurrence in HBV-related HCC patients receiving surgical resection.

## 2. Materials and Methods

### 2.1. Human Sample Collection

In this study, to investigate the association of preoperative pre-S deletions with median overall survival (OS) and recurrence-free survival (RFS), the plasma samples were obtained retrospectively from 75 HBV-related HCC patients on the day of the surgical resection they received at China Medical University Hospital (Taichung, Taiwan) from March 2004 to September 2016, under the approval of the China Medical University and Hospital Research Ethics Committee (protocol no. CMUH106-REC2-110) and were stored at −80 °C before analysis. The clinicopathological data of the patients were also collected. All research was performed in accordance with the guidelines of the 1975 Declaration of Helsinki and informed consent was obtained from all participants.

### 2.2. NGS-Based Platform for Pre-S Genotyping

The NGS-based quantitative detection of pre-S mutants in HBV-related HCC patients was carried out as described in our previous report [25]. Briefly, plasma DNA was prepared from each patient’s plasma by using the DNeasy Blood Kit (Qiagen, Valencia, CA, USA) following the manufacturer’s instructions. Next, the plasma DNA served as a template for two successive rounds of PCR amplification of the pre-S gene (comprising the pre-S1 and pre-S2 gene segments) with the high-fidelity Platinum SuperFi DNA polymerase (Invitrogen, Carlsbad, CA, USA) and specific primer pairs. The resulting pre-S gene PCR products were then directly subjected to NGS analysis by using the NextSeq 500 System supplemented with the bcl2fastq Conversion Software v2.20 (Illumina, San Diego, CA, USA) according to the manufacturer’s instructions. Finally, the pre-S deletion types, regions, and percentages were acquired by using our customized scripts. To determine the cut-off percentage of the pre-S deletions, the pre-S gene PCR products were also analyzed by thymine/adenine (TA) cloning-based pre-S genotyping.

**Figure 1:**
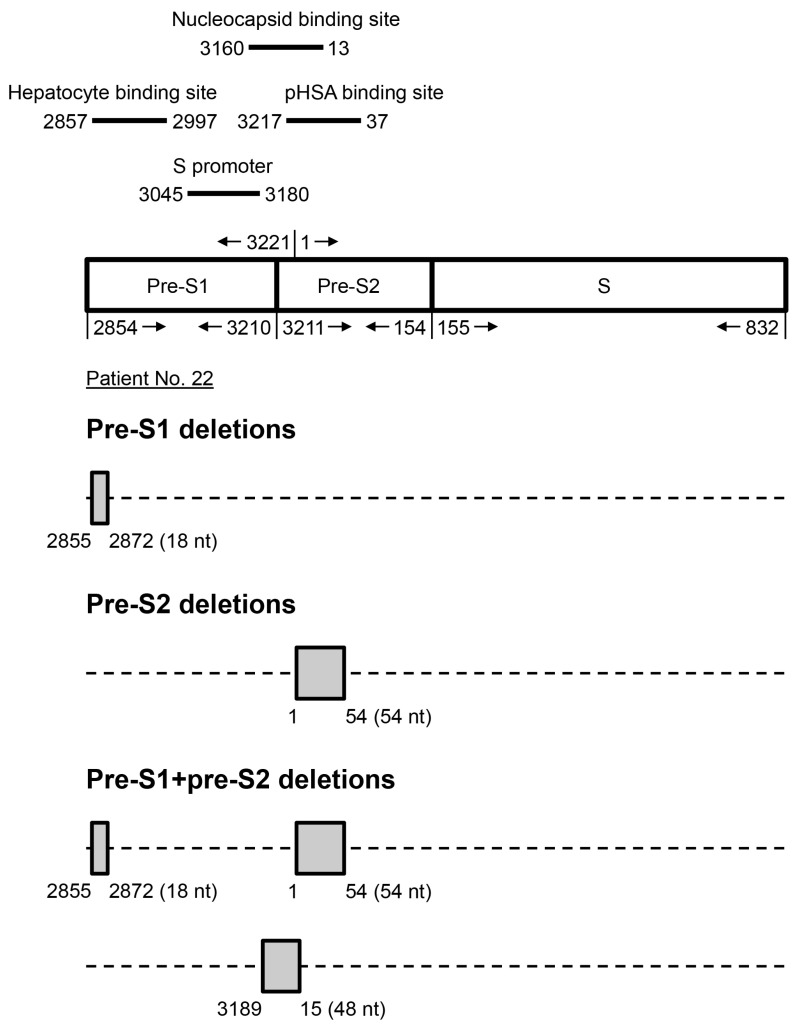
Schematic representation of HBV pre-S deletions. The HBV surface gene is composed of three gene segments: the pre-S1, pre-S2, and S gene segments. The numbers on the top and bottom of the diagram indicate the nucleotide (nt) positions of each gene segment in the circular HBV genome that starts at nt 1 and goes clockwise and ends at nt 3221. There are three types of pre-S deletions: the pre-S1, pre-S2, and pre-S1 + pre-S2 deletions. The pre-S1 deletions took place solely in the pre-S1 gene segment (nt 2854–3210); the pre-S2 deletions took place solely in the pre-S2 gene segment (nt 3211–3221, 1–154); the pre-S1 + pre-S2 deletions took place concurrently in both the pre-S1 and pre-S2 gene segments as two separate sites or as one site spanning those two gene segments. Shown are the representative deletion patterns from patient no. 22, who had all three types of pre-S deletions; the pre-S deletions with the highest frequency in each type were the pre-S1 deletion (nt 2855–2872), the pre-S2 deletion (nt 1–54), and the pre-S1 + pre-S2 deletion (nt 2855–2872, 1–54) (Table S1). Although the frequency was relatively lower, the pre-S1 + pre-S2 deletion (nt 3189-15) was shown to exemplify the deletions starting in the pre-S1 (nt 3189–3210) and continuously expanding to the pre-S2 (nt 3211–3221, 1–15) gene segment. The deletion size is shown in parentheses. Four representative functional sites encompassed within the pre-S gene are shown above the diagram. Abbreviations: pHSA, polymerized human serum albumin.

As shown in Figure 1, three types of pre-S deletions were detected: the pre-S1, pre-S2, and pre-S1 + pre-S2 deletions. The pre-S1 deletions were defined as deletions that took place solely in the pre-S1, but not in the pre-S2 gene segment; the pre-S2 deletions were defined as deletions that took place solely in the pre-S2 but not in the pre-S1 gene segment; the pre-S1 + pre-S2 deletions were defined as deletions that took place separately in both pre-S1 and pre-S2 gene segments or started in the pre-S1 and continuously expanded to the pre-S2 gene segment. Furthermore, the pre-S1 deletions and the pre-S1 + pre-S2 deletions were classified as deletions spanning the pre-S1 gene segment; the pre-S2 deletions and the pre-S1 + pre-S2 deletions were classified as deletions spanning the pre-S2 gene segment.

### 2.3. Statistical Analysis

The univariate and multivariate analyses of prognostic factors for overall and recurrence-free survival were conducted by the Cox proportional-hazards regression model. The overall and recurrence-free survival curves were estimated by the Kaplan–Meier method and compared by the log-rank test. The receiver operating characteristic (ROC) curves of prognostic factors were established for discriminating patients with HCC recurrence from those without and the area under the ROC curves (AUCs) were calculated and compared with the Hanley–McNeil test. A *p* value < 0.05 was considered significant.

## 3. Results

### 3.1. Clinicopathological Profiles of the HBV-Related HCC Patients

The clinicopathological characteristics of the 75 HBV-related HCC patients enrolled in this study are summarized in Table 1. There were 68 (91%) men and seven (9%) women. The median age of all patients was 53 years (range, 26 to 78). Sixty (80%) patients had genotype B and 15 (20%) patients had genotype C HBV infection. HBV DNA was detected in 74 (99%) patients at a median of 2.1 × 10^4^ copies/mL (range, 21.5 to 1.5 × 10^8^). Among the 65 patients with available data, all were HBsAg positive. Among the 71 patients with available data, 62 (83%) were HBV e antigen (HBeAg) negative. Tumor size was recorded for all patients with a median of 4.5 cm (range, 1.1 to 19.5). All patients received curative surgical resection. Among them, 52 (69%) patients developed HCC recurrence and 16 (21%) patients died of disease after surgery. As shown in Figure S1A,B, the OS and RFS for patients were 26.9 months (range, 6.8 to 161.1) and 11.2 months (range, 1.5 to 72.3), respectively.

### 3.2. Pre-S Genotyping and Patient Grouping by the NGS-Based Platform

The NGS- and TA cloning-based pre-S genotyping analyses were performed to detect pre-S deletions in the plasma of the 75 HBV-related HCC patients. As shown in Table S1, the TA cloning result provided information on pre-S deletion types and regions. However, the NGS result provided information on not only the pre-S deletion types and regions, but also the percentage of each of three types of pre-S deletions including the pre-S1, pre-S2, and pre-S1 + pre-S2 deletions. By matching the result of NGS with that of TA cloning, the corresponding pre-S gene DNA detected by both analyses had the lowest percentage of 4.643%, as shown by the pre-S1 deletion DNA in the patient no. 70. Therefore, we set the percentage of 4.643% as the cut-off percentage value to determine the pre-S deletion types of each patient and divide the patients into distinct pre-S genotype groups. As shown in Table 2, up to 46 of 75 (61%) patients had pre-S deletions. Among them, 15 (20%) patients had only pre-S1 deletions, six (8%) patients had only pre-S2 deletions, seven (9%) patients had both pre-S1 and pre-S2 deletions, four (5%) patients had both pre-S1 and pre-S1 + pre-S2 deletions, two (3%) patients had both pre-S2 and pre-S1 + pre-S2 deletions, and 12 (16%) patients had all three types of pre-S deletions. In addition, up to 40 (53%) and 31 (41%) of 75 patients had deletions spanning the pre-S1 and pre-S2 gene segments, respectively. Alternatively, the patients analyzed by NGS could also be classified into quarters in accordance with the percentage of any one or two or all three types of pre-S deletions from the lowest to the highest (designated I to IV, respectively).

Moreover, there was a tendency for the patients without pre-S deletions to have an extremely higher percentage of wild-type pre-S gene DNA in plasma than those with. As shown in Table S1, all 29 patients without pre-S deletions had >90% of wild-type pre-S gene DNA, whereas only four of 46 patients with pre-S deletions had >90% of wild-type pre-S gene DNA. Overall, the size of pre-S deletions with the highest frequency in each type ranged from one to 294 nucleotides in number (Table S1). The distribution of pre-S1 deletions prevalently covered the front half of the pre-S1 gene segment that partly overlapped with the hepatocyte binding site and S promoter region; the pre-S2 deletions were also predominantly distributed in the front half of the pre-S2 gene segment that was partly covered with the binding sites of nucleocapsid and polymerized human serum albumin (pHSA) (Figure 1 and Table S1).

### 3.3. Patients with Either Deletions Spanning the Pre-S2 Gene Segment or High Percentage of Pre-S2 Plus Pre-S1 + Pre-S2 Deletions as a High-Risk Population for HCC Recurrence after Surgical Resection

**Figure 2:**
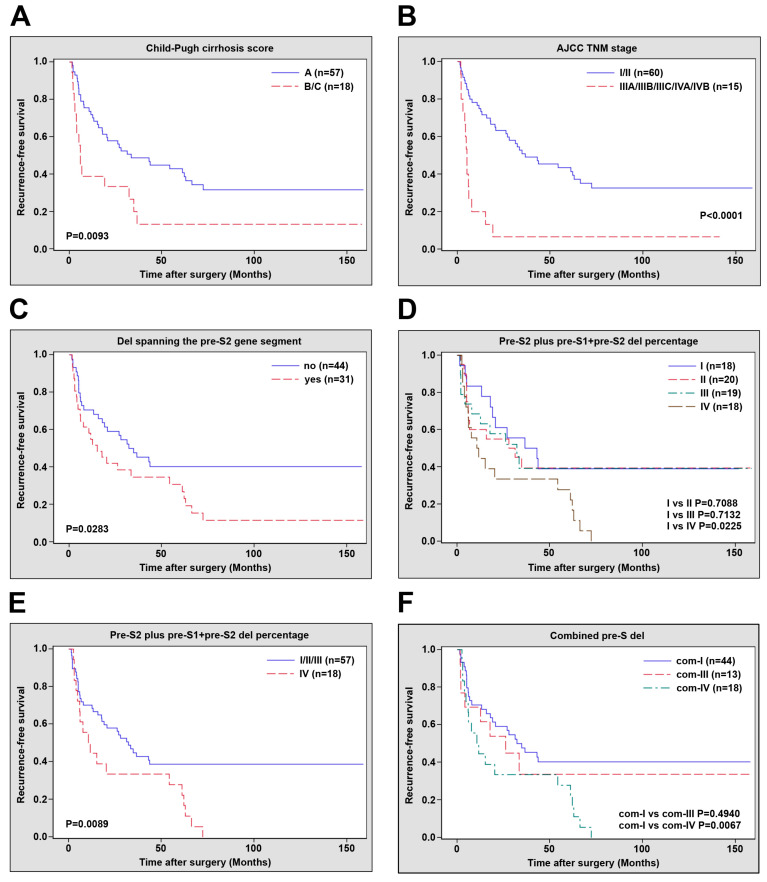
Kaplan–Meier curves of RFS differences in the 75 HBV-related HCC patients after surgical resection. (**A**) RFS in patients with Child–Pugh cirrhosis score A versus B/C. (**B**) RFS in patients with AJCC TNM stage I/II versus IIIA/IIIB/IIIC/IVA/IVB. (**C**) RFS in patients with (yes) versus without (no) deletions spanning the pre-S2 gene segment. (**D**) RFS in patients with pre-S2 plus pre-S1 + pre-S2 deletions percentage I versus II, III, or IV, respectively. (**E**) RFS in patients with pre-S2 plus pre-S1 + pre-S2 deletions percentage I/II/III versus IV. (**F**) RFS in patients with combined pre-S deletions com-I versus com-III or com-IV, respectively. RFS rate was plotted against months after surgery. *p* values and numbers (n) of patients were indicated in the plots. A *p* value < 0.05 was considered significant.

The correlations of pre-S deletion types and clinicopathological factors with OS and RFS in the 75 HBV-related HCC patients after surgical resection were examined. As shown in Table 3 and Table S2, among the clinicopathological factors analyzed, only the Child–Pugh cirrhosis score and the AJCC TNM stage were significantly and independently associated with OS (Child–Pugh cirrhosis score, hazard ratio (HR) = 2.974, 95% confidence interval (CI) 1.035 to 8.549, *p* value = 0.0430; AJCC TNM stage, HR = 3.146, 95% CI 1.041 to 9.510, *p* value = 0.0423) and RFS (Child–Pugh cirrhosis score, HR = 2.182, 95% CI 1.142 to 4.171, *p* value = 0.0182; AJCC TNM stage, HR = 3.667, 95% CI 1.853 to 7.258, *p* value = 0.0002) in patients. As shown in Figure 2A,B and Figure S2A,B, patients with Child–Pugh cirrhosis score (B/C) and AJCC TNM stage (IIIA/IIIB/IIIC/IVA/IVB) had a significantly shorter median OS (Child-Pugh cirrhosis score, 12.7 vs. 35.0 months, *p* value = 0.0324; AJCC TNM stage, 16.6 vs. 32.9 months, *p* value = 0.0306) and RFS (Child–Pugh cirrhosis score, 5.1 vs. 15.1 months, *p* value = 0.0093; AJCC TNM stage, 5.0 vs. 17.9 months, *p* value < 0.0001) than those with Child–Pugh cirrhosis score (A) and AJCC TNM stage (I/II), respectively. No significant correlations were observed between pre-S deletion types and OS in patients (Figure S2C and Table S2). However, the deletions spanning the pre-S2 gene segment, rather than the other pre-S deletion types, were defined as an independent prognostic factor for RFS in patients (HR = 2.114, 95% CI 1.203 to 3.714, *p* value = 0.0092) (Table 3). Patients with deletions spanning the pre-S2 gene segment had a significantly shorter median RFS (8.5 vs. 32.9 months, *p* value = 0.0283) than those without (Figure 2C).

Furthermore, the associations of the percentage of pre-S deletion types with OS and RFS in the 75 HBV-related HCC patients after surgical resection were investigated. As shown in Figure S2D and Table S3, there were no significant correlations between the percentage of pre-S deletion types and OS in patients. However, the highest percentage of pre-S2 plus pre-S1 + pre-S2 deletion group (IV, percentage > 24.995) showed a significantly negative prognostic impact on RFS (HR = 2.351, 95% CI 1.073 to 5.153, *p* value = 0.0328) compared with the lowest percentage of pre-S2 plus pre-S1 + pre-S2 deletion group (I, percentage ≤ 0.689) in patients (Table 4). As shown in Figure 2D, patients in group IV had significantly poorer RFS (median RFS, 11.2 vs. 19.0 months, *p* value = 0.0225) than those in the group I. At 72.3 months after surgery, all patients in group IV developed HCC recurrence, whereas seven of 18 (39%) patients in group I still did not suffer HCC recurrence. Consistently, when patients were divided into two groups, the high percentage (IV, percentage > 24.995) and low percentage (I/II/III, percentage ≤ 24.995) of pre-S2 plus pre-S1 + pre-S2 deletions, the high percentage group was significantly associated with poor RFS compared with the low percentage group of patients (HR = 2.102, 95% CI 1.148 to 3.850, *p* value = 0.0161) (Table 4). As shown in Figure 2E, when all patients in the high percentage group developed HCC recurrence after surgery, 23 of 57 (40%) patients in the low percentage group remaining did not suffer HCC recurrence.

### 3.4. Combination of Deletions Spanning the Pre-S2 Gene Segment and the Percentage of Pre-S2 Plus Pre-S1 + Pre-S2 Deletions as an Independent Prognostic Factor for HCC Recurrence after Surgical Resection

Next, the prognostic significance of the presence of deletions spanning the pre-S2 gene segment in combination with the high percentage of pre-S2 plus pre-S1 + pre-S2 deletions in the 75 HBV-related HCC patients after surgical resection was evaluated. Patients were divided into four groups based on the pre-S deletion types and percentages: com-I (44 patients), absence of deletions spanning the pre-S2 gene segment & low percentage of pre-S2 plus pre-S1 + pre-S2 deletions (I/II/III, percentage ≤ 24.995); com-II (no patients), absence of deletions spanning the pre-S2 gene segment & high percentage of pre-S2 plus pre-S1 + pre-S2 deletions (IV, percentage > 24.995); com-III (13 patients), presence of deletions spanning the pre-S2 gene segment & low percentage of pre-S2 plus pre-S1 + pre-S2 deletions; and com-IV (18 patients), presence of deletions spanning the pre-S2 gene segment & high percentage of pre-S2 plus pre-S1 + pre-S2 deletions. Because there were no patients falling into the group com-II, the prognostic significance was analyzed in the other groups compared with the group com-I. As shown in Figure S2E and Table S4, no significant differences in OS were found between the patient groups com-I and com-III as well as the groups com-I and com-IV. However, the presence of deletions spanning the pre-S2 gene segment in combination with the high percentage of pre-S2 plus pre-S1 + pre-S2 deletions (com-IV) was significantly and independently associated with poorer RFS (HR = 2.336, 95% CI 1.238 to 4.408, *p* value = 0.0088) compared with the group com-I (Table 5). As shown in Figure 2F, when all patients in the group com-IV developed HCC recurrence after surgery, 18 of 44 (41%) patients in the group com-I still did not suffer HCC recurrence. Although the median RFS for the patient group com-I versus com-IV was comparable (10.6 vs. 11.2 months), the differences in RFS between these two groups were statistically significant (*p* value = 0.0067).

**Figure 3:**
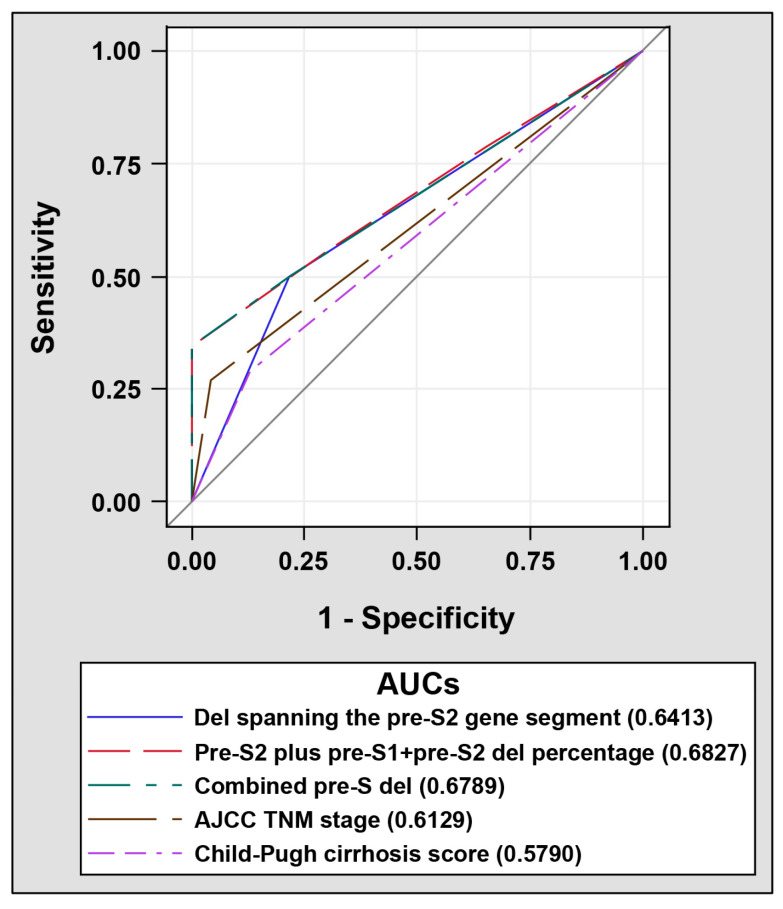
ROC curves of the selected prognostic factors in discriminating patients with HCC recurrence after surgical resection from those without. 52 patients with and 23 patients without HCC recurrence were analyzed. AUCs for selected prognostic factors, including deletions spanning the pre-S2 gene segment (solid blue line), the pre-S2 plus pre-S1 + pre-S2 deletions percentage (dashed red line), the combined pre-S deletions (dashed green line), the AJCC TNM stage (dashed brown line), and the Child-Pugh cirrhosis score (dashed purple line) were indicated in the plots.

So far, five independent prognostic factors for HCC recurrence after surgical resection were identified in the 75 HBV-related HCC patients, including the Child-Pugh cirrhosis score, the AJCC TNM stage, as well as deletions spanning the pre-S2 gene segment, the pre-S2 plus pre-S1 + pre-S2 deletions percentage, and both combined (combined pre-S deletion). The prognostic performance of these five factors was further compared. As shown in Figure 3, the pre-S2 plus pre-S1 + pre-S2 deletion percentage had the highest AUC (0.6827, 95% CI 0.5679 to 0.7975) followed by the combined pre-S deletion (0.6789, 95% CI 0.5815 to 0.7764) and then deletions spanning the pre-S2 gene segment (0.6413, 95% CI 0.5311 to 0.7515). The AUCs for the AJCC TNM stage (0.6129, 95% CI 0.5386 to 0.6872) and the Child-Pugh cirrhosis score (0.5790, 95% CI 0.4851 to 0.6729) were the lowest.

## 4. Discussion

Although surgical resection is regarded as a potentially curable treatment for HCC patients, recurrence of HCC after surgery remains a frequent event, leading to poor patient survival [4,8,9]. The presence of HBV pre-S mutants has been well demonstrated as a valuable prognostic marker for HCC recurrence in patients after surgical resection [22,23,24]. Especially, the persistence of type II GGHs, which express pre-S2 mutants, in liver tissues or the relative level of pre-S2 mutants in serum samples represents an independent risk marker for the recurrence of HBV-related HCC in patients after surgical resection, even following antiviral treatment [22,23,24]. Therefore, development of an approach that can detect pre-S mutants with high sensitivity and accuracy may hold great promise in allowing for early diagnosis and treatment of recurrent HBV-related HCC, providing survival benefits for patients undergoing surgical resection.

**Figure 4:**
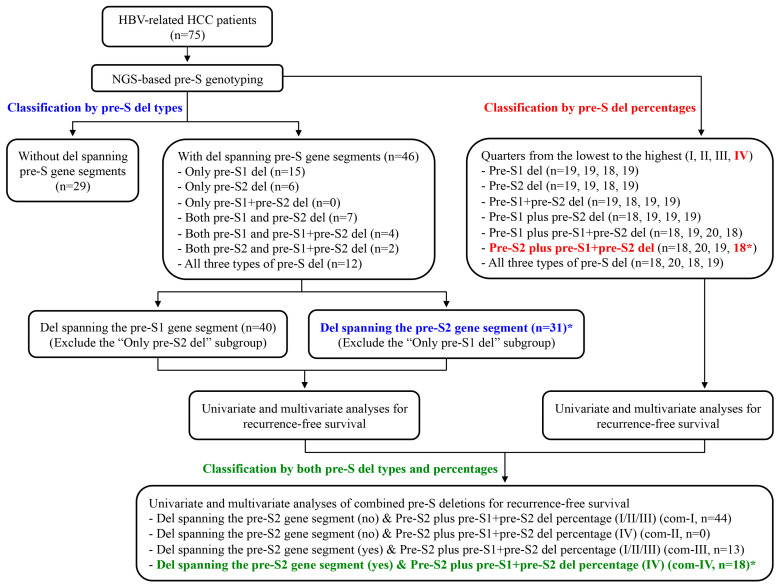
Working scheme of the NGS-based pre-S genotyping platform in the identification of patients at high risk for HCC recurrence after surgical resection. In this study, 75 HBV-related HCC patients were analyzed by the NGS-based platform for the detection of pre-S deletions. According to the types of pre-S deletions detected, patients could be classified into the groups without and with deletions spanning the pre-S gene segments. Of the two groups, the latter could be further divided into seven subgroups based on the presence of only pre-S1 deletions, only pre-S2 deletions, only pre-S1 + pre-S2 deletions, both pre-S1 and pre-S2 deletions, both pre-S1 and pre-S1 + pre-S2 deletions, both pre-S2 and pre-S1 + pre-S2 deletions, and all three types of pre-S deletions, respectively. Alternatively, the groups with deletions spanning pre-S gene segments could also be divided into two subgroups, based on the coverage of deletions on either the pre-S1 or pre-S2 gene segment. In addition, patients could be also classified into seven groups according to the percentages of pre-S1 deletions, pre-S2 deletions, pre-S1 + pre-S2 deletions, pre-S1 plus pre-S2 deletions, pre-S1 plus pre-S1 + pre-S2 deletions, pre-S2 plus pre-S1 + pre-S2 deletions, and all three types of pre-S deletions, respectively. Each of the seven groups could be further divided into quarters based on the percentages from the lowest to the highest (designated I to IV, respectively). By univariate and multivariate analyses, either the presence of deletions spanning the pre-S2 gene segment or the highest percentage of pre-S2 plus pre-S1 + pre-S2 deletions was identified as an independent prognostic factor for HCC recurrence after surgical resection. Furthermore, patients could be classified into four groups according to the absence (no)/presence (yes) of deletions spanning the pre-S2 gene segment and the low (I/II/III)/high (IV) percentage of pre-S2 plus pre-S1 + pre-S2 deletions (designated com-I to com-IV, respectively). Through univariate and multivariate analyses, the presence of deletions spanning the pre-S2 gene segment in combination with the high percentage of pre-S2 plus pre-S1 + pre-S2 deletions was also validated as an independent factor for predicting HCC recurrence after surgical resection. Numbers (n) of patients in each group were indicated in parentheses. The patient groups at high risk for HCC recurrence were highlighted with asterisks and different colors according to the classification based on the pre-S deletion types (blue), percentages (red), and both types and percentages (green). Abbreviations: del, deletion; com, combined.

Compared with current approaches that can provide only qualitative and semi-quantitative detection results of pre-S deletions [18,19,22], the NGS-based platform developed by our group can detect pre-S deletions quantitatively, accompanied with higher sensitivity and fidelity [25,26]. As a result, by means of this platform, the percentage of each of the three types of pre-S deletions including the pre-S1, pre-S2, and pre-S1 + pre-S2 deletions in plasma samples of HBV-related HCC patients could be well determined and patients could then be classified into distinctly defined groups according to not only the types, but also the percentages of pre-S deletions detected (Figure 4). First, on the basis of the types or percentages of pre-S deletions, we identified that either the presence of deletions spanning the pre-S2 gene segment (cut-off percentage of 4.643) or the high percentage of pre-S2 plus pre-S1 + pre-S2 deletions (percentage > 24.995) was an independent prognostic factor for HCC recurrence after surgical resection. Next, we validated that the combination of these two pre-S deletion factors (both the types and percentages) was also an independent factor associated with HCC recurrence after surgery. Furthermore, we demonstrated that the presence of deletions spanning the pre-S2 gene segment and the high percentage of pre-S2 plus pre-S1 + pre-S2 deletions, either alone or in combination, had superior prognostic efficiency to other independent factors (the Child–Pugh cirrhosis score and the AJCC TNM stage) in predicting HCC recurrence in patients after surgical resection. Therefore, we suggest that upon analysis by the established NGS-based platform, patients with either or both deletions spanning the pre-S2 gene segment and the high percentage of pre-S2 plus pre-S1 + pre-S2 deletions are regarded as a high-risk population for HCC recurrence after surgical resection. Our NGS-based detection results consistently emphasize the significant association of pre-S2 mutants with HBV-related HCC recurrence after surgical resection.

A variety of prognostic markers have been proposed for HCC recurrence after surgical resection. Most of them are host oncoproteins and tumor suppressors in liver tissues [27]. For example, high level of oncoproteins c-Myc and β-catenin expression is associated with shorter RFS in HCC patients [28,29]. In addition, the combination of low level of tumor suppressor phosphatase and tensin homology and high level of oncoproteins proliferating cell nuclear antigen and p53 expression is correlated with poorer RFS in HCC patients [30]. Although these markers display significant prognostic values for HCC prognosis, their clinical utilization is severely hindered by the need and risk of invasive tissue biopsy-based detection procedures. In this study, we evaluated pre-S mutants (both the deletion types and percentages) as a plasma biomarker for predicting HCC recurrence after surgical resection. Compared with tissues, plasma is a more readily accessible specimen source and is commonly used as the so-called noninvasive liquid biopsy. Therefore, the NGS-based detection of pre-S mutants in plasma may hold a greater promise in clinical application, especially for patients unsuitable for needle biopsy of the liver. In addition, a high level of preoperative serum HBV DNA has been reported to be associated with postoperative HCC recurrence though the cut-off value for serum HBV DNA level and the postoperative follow-up time varies between different studies [31,32,33]. Furthermore, antiviral therapy, which suppresses HBV replication, thus decreasing serum HBV DNA level, has been shown to significantly improve RFS in HCC patients after surgical resection [34,35]. However, the beneficial effect of antiviral therapy is exclusively found in patients with a high level of preoperative serum HBV DNA and depends on continuous antiviral treatments after surgery [36]. In this study, neither the serum HBV DNA level nor antiviral therapy was observed to be associated with RFS in HCC patients; the former might be possibly because a different cut-off value was needed to divide patients, and the latter because not all patients enrolled in this study had high serum HBV DNA level and received antiviral therapy after surgery. Instead, the pre-S deletions detected by the NGS-based platform were evaluated as an independent prognostic factor for HCC recurrence. Consistent with our results, the type II GGHs harboring pre-S2 mutants in liver tissues have been shown to resist antiviral therapy, regardless of the decreased levels of serum HBV DNA, and is significantly implicated in the recurrence of HCC in patients after surgical resection [23]. Our results thus suggest that in some patient cohorts, pre-S mutants may have potentially better prognostic performance for HCC recurrence than other viral factors.

In the HBV replication cycle, the relaxed circular form of HBV DNA after entering the host hepatocytes will be converted into the covalently closed circular DNA that serves as the template for the production of HBV pregenomic RNA and gene products, followed by the assembly and release of HBV virions [37,38,39]. However, during the replication cycle, integration of HBV DNA into the host hepatocyte genome is a frequent event, potentially leading to insertional mutagenesis, genomic instability, and persistent viral gene expression that may promote hepatocyte transformation and eventually the development of HCC [40,41,42]. HBV DNA integrations occur in the early stage of chronic HBV infection and can be detected in about 30% of adjacent non-tumor cells and up to 90% of HCC cells [43,44]. Therefore, the HBV DNA fragments obtained from blood samples of HBV-related HCC patients may be derived from two sources, one associated with HBV virions and the other released from dead hepatocytes or HCC cells that harbor HBV DNA integrations. A study by Jia et al. compared the deletion patterns and incidences of pre-S gene in matched serum, adjacent non-tumor tissues, and tumor tissues from 40 HBV-related HCC patients, and showed that deletions in the pre-S gene among the different samples exhibited consistent distribution and similar abundance [45]. This finding supports that pre-S mutants may originate from the integrated HBV DNA that contains pre-S deletions in HBV-infected hepatocytes and HCC cells, possibly explaining the association of pre-S mutants with HCC recurrence in patients after surgical resection, even after receiving postoperative antiviral therapy. Further investigations are still needed to compare the profiles of pre-S deletions in matched blood and liver tissue specimens obtained from HBV-related HCC patients before and after surgical resection in a long-term follow-up and evaluate their clinical and pathological implications in HCC recurrence.

## 5. Conclusions

In this study, we demonstrated the NGS-based quantitative detection of pre-S mutants in plasma as a powerful approach for identifying the patients at high risk for HBV-related HCC recurrence after surgical resection. The presence of deletions spanning the pre-S2 gene segment and the high percentage of pre-S2 plus pre-S1 + pre-S2 deletions, either alone or in combination, represents a promising noninvasive biomarker for predicting HCC recurrence in patients undergoing surgical resection.

## Figures and Tables

**Table 1:** Clinicopathological characteristics of 75 HBV-related HCC patients enrolled in this study.

Characteristics	No. of Patients	Median (Range)
Age (years)	75	53 (26–78)
>50	48	60 (51–78)
≤50	27	43 (26–50)
Gender (men/women)	68/7	
Smoking (yes/no)	31/44	
Alcohol (yes/no)	29/46	
HBsAg (positive/negative/NA)	65/0/10	
HBeAg (positive/negative/NA)	9/62/4	
HBV genotype (B/C)	60/15	
HBV DNA (IU/mL) (20–1.7 × 10^8^/<20) ^a^	74/1	2.1 × 10^4^ (21.5–1.5 × 10^8^) ^c^
>1 × 10^4^	42	4.3 × 10^5^ (1.2 × 10^4^–1.5 × 10^8^)
≤1 × 10^4^	32	8.4 × 10^2^ (21.5–9.3 × 10^3^)
Albumin (g/dL)	75	3.7 (1.2–4.9)
>3.8	30	4.2 (3.9–4.9)
≤3.8	45	3.3 (1.2–3.8)
AST (U/L)	75	60 (14–1052)
>34	61	79 (35–1052)
≤34	14	27 (14–34)
ALT (U/L)	75	55 (13–1338)
>40	50	96.5 (41–1338)
≤40	25	31 (13–40)
AFP (ng/mL) (≤54,000/>54,000) ^b^	71/4	26.7 (1.8–36,600.0) ^d^
>400	28	1920 (461.7–36,600.0)
≤400	47	13.8 (1.8–271.0)
Tumor size (cm)	75	4.5 (1.1–19.5)
>5	37	10.0 (5.5–19.5)
≤5	38	2.4 (1.1–4.5)
Tumor encapsulation (yes/no/NA)	42/20/13	
Lymph node involvement (yes/no)	8/67	
Portal vein thrombosis (yes/no)	5/70	
Vascular invasion (yes/no)	27/48	
Distant metastasis (yes/no)	8/67	
Steatosis grade (0/1/2/3/NA)	14/10/1/0/50	
Metavir inflammation score (0/1/2/3/NA)	4/35/5/0/31	
Ishak fibrosis score (0/1/2/3/4/5/6/NA)	5/13/12/8/3/4/11/19	
Child-Pugh cirrhosis score (A/B/C)	57/16/2	
CLIP score (0/1/2/3/4/5/6)	33/23/10/8/1/0/0	
Tumor differentiation grade (1/2/3/4)	2/36/36/1	
BCLC stage (A/B/C/D)	38/29/7/1	
AJCC TNM stage (I/II/IIIA/IIIB/IIIC/IVA/IVB)	40/20/7/5/3/0/0	
Antiviral therapy after surgery (yes/no)	40/35	
HCC recurrence after surgery (month) (yes/no)	52/23	11.2 (1.5–72.3) ^e^
Survival after surgery (month) (dead/alive)	16/59	26.9 (6.8–161.1) ^f^

^a^ HBV DNA was measured with a detection range of 20 to 1.7 × 10^8^ IU/mL. ^b^ AFP was measured with the highest detection limit of 54,000 ng/mL. ^c,d^ Only data within the detection range were analyzed. ^e^ Shown was the time to recurrence after surgery. ^f^ Shown was survival time in patients who died after surgery. Abbreviations: HBV, hepatitis B virus; HCC, hepatocellular carcinoma; HBeAg, hepatitis B e antigen; NA, not available; AST, aspartate aminotransferase; ALT, alanine aminotransferase; AFP, alpha-fetoprotein; CLIP, Cancer of the Liver Italian Program; BCLC, Barcelona Clinic Liver Cancer; AJCC, American Joint Committee on Cancer; TNM, tumor-node-metastasis.

**Table 2:** Summary of NGS-based pre-S genotyping result in 75 HBV-related HCC patients.

Pre-S Del Type of Patients ^a^	No. of Patients (%) ^b^
Total patients	75 (100)
Patients without del spanning pre-S gene segments	29 (39)
Patients with del spanning pre-S gene segments	46 (61)
Patients with only pre-S1 del	15 (20)
Patients with only pre-S2 del	6 (8)
Patients with only pre-S1 + pre-S2 del	0 (0)
Patients with both pre-S1 and pre-S2 del	7 (9)
Patients with both pre-S1 and pre-S1 + pre-S2 del	4 (5)
Patients with both pre-S2 and pre-S1 + pre-S2 del	2 (3)
Patients with all three types of pre-S del	12 (16)
Patients with del spanning the pre-S1 gene segment	40 (53)
Patients with del spanning the pre-S2 gene segment	31 (41)
**Pre-S Del Percentage of Patients**	**No. of Patients (%)**
Pre-S1 del percentage ^c^	75 (100)
I (≤1.895) ^d^	19 (25)
II (>1.895)	19 (25)
III (>5.629)	18 (24)
IV (>28.270)	19 (25)
Pre-S2 del percentage	75 (100)
I (≤0.643)	19 (25)
II (>0.643)	19 (25)
III (>1.824)	18 (24)
IV (>13.666)	19 (25)
Pre-S1 + pre-S2 del percentage	75 (100)
I (≤0.015)	19 (25)
II (>0.015)	18 (24)
III (>0.442)	19 (25)
IV (>2.975)	19 (25)
Pre-S1 plus pre-S2 del percentage	75 (100)
I (≤2.725)	18 (24)
II (>2.725)	19 (25)
III (>18.539)	19 (25)
IV (>49.938)	19 (25)
Pre-S1 plus pre-S1 + pre-S2 del percentage	75 (100)
I (≤2.043)	18 (24)
II (>2.043)	19 (25)
III (>6.332)	20 (27)
IV (>44.201)	18 (24)
Pre-S2 plus pre-S1 + pre-S2 del percentage	75 (100)
I (≤0.689)	18 (24)
II (>0.689)	20 (27)
III (>2.109)	19 (25)
IV (>24.995)	18 (24)
All three types of pre-S del percentage	75 (100)
I (≤2.828)	18 (24)
II (>2.828)	20 (27)
III (>21.666)	18 (24)
IV (>58.035)	19 (25)

^a^ Pre-S del type of patients was determined by a cut-off percentage of 4.643. ^b^ Percentage of patients was rounded off to the nearest integer. ^c^ Patients were divided into quarters of the distribution of indicated type of pre-S del percentage from the lowest to the highest (designated I to IV, respectively). ^d^ Threshold percentage of indicated pre-S del type for each quarter was shown in parenthesis. Abbreviations: del, deletion.

**Table 3:** Univariate and multivariate analyses of pre-S deletion type for recurrence-free survival in 75 HBV-related HCC patients.

Characteristics	Univariate Analysis			Multivariate Analysis		
	HR	95% CI	*p* Value	HR	95% CI	*p* Value
Age (years) (>50 vs. ≤50)	0.951	0.532–1.700	0.8666			
Gender (men vs. women)	1.043	0.414–2.627	0.9284			
Smoking (yes vs. no)	0.886	0.503–1.560	0.6750			
Alcohol (yes vs. no)	0.884	0.494–1.580	0.6773			
HBsAg (positive vs. negative) ^a^						
HBeAg (positive vs. negative) ^b^	1.234	0.523–2.910	0.6307			
HBV genotype (B vs. C)	0.583	0.304–1.117	0.1040			
HBV DNA (IU/mL) (>1 × 10^4^ vs. ≤1 × 10^4^) ^c^	1.645	0.934–2.895	0.0846			
Albumin (g/dL) (>3.8 vs. ≤3.8)	0.551	0.288–1.092	0.0585			
AST (U/L) (>34 vs. ≤34)	0.865	0.444–1.684	0.6691			
ALT (U/L) (>40 vs. ≤40)	0.797	0.456–1.394	0.4267			
AFP (ng/mL) (>400 vs. ≤400)	1.305	0.745–2.285	0.3524			
Tumor size (cm) (>5 vs. ≤5)	1.490	0.863–2.572	0.1525			
Tumor encapsulation (yes vs. no) ^d^	0.901	0.474–1.713	0.7508			
Lymph node involvement (yes vs. no)	0.333	0.104–1.071	0.0652			
Portal vein thrombosis (yes vs. no)	1.668	0.600–4.633	0.3264			
Vascular invasion (yes vs. no)	1.677	0.962–2.924	0.0681			
Distant metastasis (yes vs. no)	2.259	0.999–5.101	0.0502			
Steatosis grade (2/3 vs. 0/1) ^e^	3.473	0.418–28.879	0.2493			
Metavir inflammation score (2/3 vs. 0/1) ^f^	0.731	0.256–2.088	0.5583			
Ishak fibrosis score (4/5/6 vs. 0/1/2/3) ^g^	1.261	0.670–2.373	0.4714			
Child-Pugh cirrhosis score (B/C vs. A)	2.189	1.195–4.013	0.0112 *	2.182	1.142–4.171	0.0182 *
CLIP score (4/5/6 vs. 0/1/2/3)	2.426	0.328–17.911	0.3850			
Tumor differentiation (3/4 vs. 1/2)	1.246	0.722–2.150	0.4288			
BCLC stage (C/D vs. A/B)	1.927	0.867–4.284	0.1077			
AJCC TNM stage (IIIA/IIIB/IIIC/IVA/IVB vs. I/II)	4.048	2.123–7.719	<0.0001 ***	3.667	1.853–7.258	0.0002 ***
Antiviral therapy after surgery (yes vs. no)	1.176	0.674–2.051	0.5684			
Del spanning pre-S gene segments (yes vs. no)	1.315	0.742–2.332	0.3477			
Only pre-S1 del (yes vs. no)	0.590	0.277–1.257	0.1715			
Only pre-S2 del (yes vs. no)	1.421	0.564–3.580	0.4557			
Only pre-S1 + pre-S2 del (yes vs. no) ^h^						
Both pre-S1 and pre-S2 del (yes vs. no)	1.260	0.453–3.505	0.6573			
Both pre-S1 and pre-S1 + pre-S2 del (yes vs. no)	2.460	0.876–6.909	0.0876			
Both pre-S2 and pre-S1 + pre-S2 del (yes vs. no)	1.621	0.393–6.690	0.5042			
All three types of pre-S del (yes vs. no)	1.352	0.694–2.632	0.3755			
Del spanning the pre-S1 gene segment (yes vs. no)	1.165	0.673–2.015	0.5859			
Del spanning the pre-S2 gene segment (yes vs. no)	1.825	1.058–3.149	0.0307 *	2.114	1.203–3.714	0.0092 **

^a^ There were no patients negative for HBsAg for analysis. ^b^ Only 71 patients with available data were analyzed. ^c^ Only 74 patients with available data were analyzed. ^d^ Only 62 patients with available data were analyzed. ^e^ Only 25 patients with available data were analyzed. ^f^ Only 44 patients with available data were analyzed. ^g^ Only 56 patients with available data were analyzed. ^h^ There were no patients with only pre-S1 + pre-S2 del for analysis. * *p* value < 0.05; ** *p* value < 0.01; *** *p* value < 0.001. Abbreviations: HR, hazard ratio; CI, confidence interval; del, deletion; vs., versus.

**Table 4:** Univariate and multivariate analyses of pre-S deletion percentage for recurrence-free survival in 75 HBV-related HCC patients.

Characteristics	Univariate Analysis			Multivariate Analysis		
	HR	95% CI	*p* Value	HR	95% CI	*p* Value
Child–Pugh cirrhosis score (B/C vs. A)	2.189	1.195–4.013	0.0112 *	2.148	1.113–4.147	0.0227 *^,c^
				2.123	1.114–4.045	0.0221 *^,d^
AJCC TNM stage (IIIA/IIIB/IIIC/IVA/IVB vs. I/II)	4.048	2.123–7.719	<0.0001 ***	3.225	1.600–6.500	0.0011 **^,c^
				3.207	1.605–6.410	0.0010 **^,d^
Pre-S1 del percentage ^a^						
II (>1.895) vs. I (≤1.895) ^b^	0.752	0.342–1.653	0.4778			
III (>5.629) vs. I (≤1.895)	1.116	0.524–2.378	0.7760			
IV (>28.270) vs. I (≤1.895)	0.984	0.455–2.128	0.9670			
Pre-S2 del percentage						
II (>0.643) vs. I (≤0.643)	1.091	0.479–2.483	0.8357			
III (>1.824) vs. I (≤0.643)	1.272	0.560–2.887	0.5658			
IV (>13.666) vs. I (≤0.643)	2.020	0.971–4.202	0.0600			
Pre-S1 + pre-S2 del percentage						
II (>0.015) vs. I (≤0.015)	0.501	0.207–1.212	0.1251			
III (>0.442) vs. I (≤0.015)	1.109	0.513–2.396	0.7927			
IV (>2.975) vs. I (≤0.015)	1.497	0.733–3.058	0.2687			
Pre-S1 plus pre-S2 del percentage						
II (>2.725) vs. I (≤2.725)	0.873	0.391–1.946	0.7392			
III (>18.539) vs. I (≤2.725)	1.029	0.474–2.233	0.9431			
IV (>49.938) vs. I (≤2.725)	1.220	0.562–2.646	0.6152			
Pre-S1 plus pre-S1 + pre-S2 del percentage						
II (>2.043) vs. I (≤2.043)	0.805	0.361–1.797	0.5965			
III (>6.332) vs. I (≤2.043)	1.035	0.483–2.216	0.9300			
IV (>44.201) vs. I (≤2.043)	1.162	0.529–2.553	0.7093			
Pre-S2 plus pre-S1 + pre-S2 del percentage						
II (>0.689) vs. I (≤0.689)	1.112	0.490–2.526	0.7996	1.196	0.524–2.732	0.6713
III (>2.109) vs. I (≤0.689)	1.102	0.477–2.547	0.8208	1.170	0.498–2.744	0.7190
IV (>24.995) vs. I (≤0.689)	2.265	1.067–4.811	0.0333 *	2.351	1.073–5.153	0.0328 *^c^
IV (>24.995) vs. I/II/III (≤24.995) ^c^	2.118	1.191–3.765	0.0106 *	2.102	1.148–3.850	0.0161 *^d^
All three types of pre-S del percentage						
II (>2.828) vs. I (≤2.828)	0.688	0.302–1.564	0.3720			
III (>21.666) vs. I (≤2.828)	0.819	0.373–1.802	0.6202			
IV (>58.035) vs. I (≤2.828)	1.759	0.827–3.739	0.1422			

^a^ Patients were divided into quarters of the distribution of indicated type of pre-S del percentage from the lowest to the highest (designated I to IV, respectively). ^b^ Threshold percentage of indicated pre-S del type for each quarter was shown in parenthesis. ^c,d^ Multivariate analysis was performed between these characteristics accordingly. * *p* value < 0.05; ** *p* value < 0.01; *** *p* value < 0.001. Abbreviations: HR, hazard ratio; CI, confidence interval; del, deletion.

**Table 5:** Univariate and multivariate analyses of combined pre-S deletion for recurrence-free survival in 75 HBV-related HCC patients.

Characteristics	Univariate Analysis			Multivariate Analysis		
	HR	95% CI	*p* Value	HR	95% CI	*p* Value
Child-Pugh cirrhosis score (B/C vs. A)	2.189	1.195–4.013	0.0112 *	2.213	1.154–4.244	0.0168 *
AJCC TNM stage (IIIA/IIIB/IIIC/IVA/IVB vs. I/II)	4.048	2.123–7.719	<0.0001 ***	3.440	1.689–7.005	0.0007 ***
Combined pre-S del ^a^						
com-II ^b^ vs. com-I						
com-III vs. com-I	1.294	0.585–2.863	0.5242	1.764	0.780–3.986	0.1726
com-IV vs. com-I	2.237	1.222–4.097	0.0091 **	2.336	1.238–4.408	0.0088 **

^a^ Patients were divided into four groups based on pre-S del types and percentages: com-I, del spanning the pre-S2 gene segment (no) and pre-S2 plus pre-S1 + pre-S2 del percentage (I/II/III); com-II, del spanning the pre-S2 gene segment (no) & pre-S2 plus pre-S1 + pre-S2 del percentage (IV); com-III, del spanning the pre-S2 gene segment (yes) & pre-S2 plus pre-S1 + pre-S2 del percentage (I/II/III); and com-IV, del spanning the pre-S2 gene segment (yes) & pre-S2 plus pre-S1 + pre-S2 del percentage (IV). ^b^ There were no patients falling into the com-II group for analysis. * *p* value < 0.05; ** *p* value < 0.01; *** *p* value < 0.001. Abbreviations: HR, hazard ratio; CI, confidence interval; del, deletion; com, combined.

## Supplementary Materials

The following are available online at https://www.mdpi.com/1999-4915/12/8/796/s1, Figure S1: Kaplan-Meier curves of OS and RFS differences in the 75 HBV-related HCC patients after surgical resection, Figure S2: Kaplan-Meier curves of OS differences in the 75 HBV-related HCC patients after surgical resection, Table S1: List of the pre-S genotyping results by TA cloning- and NGS-based analyses in 75 HBV-related HCC patients, Table S2: Univariate and multivariate analyses of pre-S deletion type for overall survival in 75 HBV-related HCC patients, Table S3: Univariate and multivariate analyses of pre-S deletion percentage for overall survival in 75 HBV-related HCC patients, Table S4: Univariate and multivariate analyses of combined pre-S deletion for overall survival in 75 HBV-related HCC patients.
