# Supplementary material for: Next-Generation Sequencing-Based Quantitative Detection of Hepatitis B Virus Pre-S Mutants in Plasma Predicts Hepatocellular Carcinoma Recurrence
Source: Viruses. 2020 Jul 24;12(8):796. doi: 10.3390/v12080796 (PMC7472021; doi:10.3390/v12080796)
Supplement: Supplementary file 1 [file viruses-12-00796-s001.zip › viruses-850144-for conversion-suppl_/Supporting figure legends.pdf]

Supporting figure legends

Figure S1. Kaplan-Meier curves of OS and RFS differences in the 75 HBV-related HCC patients after surgical resection. (A) OS in all patients without grouping. (B) RFS in all patients without grouping. OS and RFS rate was plotted against months after surgery.

Figure S2. Kaplan-Meier curves of OS differences in the 75 HBV-related HCC patients after surgical resection. (A) OS in patients with Child-Pugh cirrhosis score A versus B/C. (B) OS in patients with AJCC TNM stage I/II versus IIIA/IIIB/IIIC/IVA/IVB. (C) OS in patients with (yes) versus without (no) deletions spanning the pre-S2 gene segment. (D) OS in patients with pre-S2 plus pre-S1+pre-S2 deletions percentage I versus II, III, or IV. (E) OS in patients with combined pre-S deletions com-I versus com-III or com-IV. OS rate was plotted against months after surgery. *P* values and numbers of patients were indicated in the plots. A *P* value < 0.05 was considered significant.
