# Supplementary material for: Next-Generation Sequencing-Based Quantitative Detection of Hepatitis B Virus Pre-S Mutants in Plasma Predicts Hepatocellular Carcinoma Recurrence
Source: Viruses. 2020 Jul 24;12(8):796. doi: 10.3390/v12080796 (PMC7472021; doi:10.3390/v12080796)
Supplement: Supplementary file 1 [file viruses-12-00796-s001.zip › viruses-850144-for conversion-suppl_/Table S1,2,3,4.pdf]

Table S1. List of the pre-S genotyping results by TA cloning- and NGS-based analyses in 75 HBV-related HCC patients

Patient No.	TA Cloning Result[a]	NGS Result (Pre-S Del Type (%))[b]	NGS Result (Pre-S Del Region (%))[c]
1	pre-S1 del (nt 2855-2872)	1. **pre-S1 del (92.118)**[d]	1. pre-S1 del (nt 2855-2872) (90.102)
		2. **wild-type (7.278)**	2. wild-type (7.278)
		3. pre-S2 del (0.372)	3. pre-S2 del (nt 1-28) (0.180)
		4. pre-S1+pre-S2 del (0.231)	4. pre-S1+pre-S2 del (nt 2855-2872, 1-111) (0.027)
		I. pre-S1 plus pre-S2 del (92.490)[e]	
		II. pre-S1 plus pre-S1+pre-S2 del (92.349)	
		III. pre-S2 plus pre-S1+pre-S2 del (0.603)	
		IV. all three types of pre-S del (92.722)	
2	1. wild-type	1. **pre-S1 del (75.241)**	1. pre-S1 del (nt 2910-3089) (48.977)
	2. pre-S1 del (nt 2910-3089)	2. **wild-type (22.338)**	2. wild-type (22.338)
		3. pre-S2 del (1.891)	3. pre-S2 del (nt 1-54) (1.579)
		4. pre-S1+pre-S2 del (0.530)	4. pre-S1+pre-S2 del (nt 2910-3089, 1-57) (0.277)
		I. pre-S1 plus pre-S2 del (77.132)	
		II. pre-S1 plus pre-S1+pre-S2 del (75.771)	
		III. pre-S2 plus pre-S1+pre-S2 del (2.421)	
		IV. all three types of pre-S del (77.662)	
3	wild-type	1. **pre-S1 del (76.228)**	1. pre-S1 del (nt 3110-3127) (24.869)
		2. **wild-type (12.583)**	2. wild-type (12.583)
		3. **pre-S2 del (10.622)**	3. pre-S2 del (nt 1-57) (10.000)
		4. pre-S1+pre-S2 del (0.567)	4. pre-S1+pre-S2 del (nt 2855-2872, 1-57) (0.490)
		I. pre-S1 plus pre-S2 del (86.850)	
		II. pre-S1 plus pre-S1+pre-S2 del (76.795)	
		III. pre-S2 plus pre-S1+pre-S2 del (11.189)	
		IV. all three types of pre-S del (87.417)	

4	wild-type	1. **wild-type (71.590)**<br>2. **pre-S1 del (28.270)**<br>3. pre-S2 del (0.129)<br>4. pre-S1+pre-S2 del (0.011)<br>I. pre-S1 plus pre-S2 del (28.399)<br>II. pre-S1 plus pre-S1+pre-S2 del (28.281)<br>III. pre-S2 plus pre-S1+pre-S2 del (0.140)<br>IV. all three types of pre-S del (28.410)	1. wild-type (71.590)<br>2. pre-S1 del (nt 2854-3147) (23.961)<br>3. pre-S2 del (nt 3211-3216) (0.048)<br>4. pre-S1+pre-S2 del (nt 2954-3097, 1-54) (0.002)
5	pre-S2 del (nt 24-50)	1. **wild-type (58.461)**<br>2. **pre-S2 del (37.194)**<br>3. pre-S1 del (2.939)<br>4. pre-S1+pre-S2 del (1.407)<br>I. pre-S1 plus pre-S2 del (40.133)<br>II. pre-S1 plus pre-S1+pre-S2 del (4.346)<br>III. pre-S2 plus pre-S1+pre-S2 del (38.601)<br>IV. all three types of pre-S del (41.539)	1. wild-type (58.461)<br>2. pre-S2 del (nt 24-50) (33.801)<br>3. pre-S1 del (nt 2880-3146) (0.646)<br>4. pre-S1+pre-S2 del (nt 2880-3146, 24-50) (0.355)
6	wild-type	1. **wild-type (96.592)**<br>2. pre-S1 del (2.906)<br>3. pre-S2 del (0.469)<br>4. pre-S1+pre-S2 del (0.033)<br>I. pre-S1 plus pre-S2 del (3.375)<br>II. pre-S1 plus pre-S1+pre-S2 del (2.939)<br>III. pre-S2 plus pre-S1+pre-S2 del (0.502)<br>IV. all three types of pre-S del (3.408)	1. wild-type (96.592)<br>2. pre-S1 del (nt 3026-3205) (0.604)<br>3. pre-S2 del (nt 1-12) (0.233)<br>4. pre-S1+pre-S2 del (nt 2854-2979, 6-134) (0.008)
7	wild-type	1. **wild-type (99.000)**<br>2. pre-S1 del (0.927)	1. wild-type (99.000)<br>2. pre-S1 del (nt 3106-3129) (0.294)

		3. pre-S2 del (0.062)	3. pre-S2 del (nt 1-54) (0.031)
		4. pre-S1+pre-S2 del (0.012)	4. pre-S1+pre-S2 del (nt 2855-2972, 1-54) (0.008)
		I. pre-S1 plus pre-S2 del (0.989)	
		II. pre-S1 plus pre-S1+pre-S2 del (0.939)	
		III. pre-S2 plus pre-S1+pre-S2 del (0.074)	
		IV. all three types of pre-S del (1.000)	
8	wild-type	1. **wild-type (93.746)**	1. wild-type (93.746)
		2. **pre-S1 del (5.846)**	2. pre-S1 del (nt 3026-3205) (2.417)
		3. pre-S2 del (0.389)	3. pre-S2 del (nt 3211-3213) (0.135)
		4. pre-S1+pre-S2 del (0.018)	4. pre-S1+pre-S2 del (nt 2856-2873, 1-21) (0.004)
		I. pre-S1 plus pre-S2 del (6.235)	
		II. pre-S1 plus pre-S1+pre-S2 del (5.864)	
		III. pre-S2 plus pre-S1+pre-S2 del (0.407)	
		IV. all three types of pre-S del (6.254)	
9	wild-type	1. **pre-S1+pre-S2 del (46.237)**	1. pre-S1+pre-S2 del (nt 2956-3126, 1-9) (24.002)
		2. **pre-S2 del (26.927)**	2. pre-S2 del (nt 1-54) (23.226)
		3. **pre-S1 del (14.368)**	3. pre-S1 del (nt 2944-3075) (5.140)
		4. **wild-type (12.467)**	4. wild-type (12.467)
		I. pre-S1 plus pre-S2 del (41.295)	
		II. pre-S1 plus pre-S1+pre-S2 del (60.605)	
		III. pre-S2 plus pre-S1+pre-S2 del (73.164)	
		IV. all three types of pre-S del (87.533)	
10	wild-type	1. **wild-type (97.851)**	1. wild-type (97.851)
		2. pre-S1 del (2.037)	2. pre-S1 del (nt 3026-3205) (1.109)
		3. pre-S2 del (0.108)	3. pre-S2 del (nt 3211-3213) (0.049)
		4. pre-S1+pre-S2 del (0.005)	4. pre-S1+pre-S2 del (nt 2855-2872, 1-54) (0.003)

		I. pre-S1 plus pre-S2 del (2.145)	
		II. pre-S1 plus pre-S1+pre-S2 del (2.042)	
		III. pre-S2 plus pre-S1+pre-S2 del (0.113)	
		IV. all three types of pre-S del (2.149)	
11	wild-type	1. **wild-type (98.964)**	1. wild-type (98.964)
		2. pre-S1 del (0.943)	2. pre-S1 del (nt 2854-2970) (0.125)
		3. pre-S2 del (0.084)	3. pre-S2 del (nt 1-9) (0.028)
		4. pre-S1+pre-S2 del (0.009)	4. pre-S1+pre-S2 del (nt 2954-3097, 1-13) (0.009)
		I. pre-S1 plus pre-S2 del (1.027)	
		II. pre-S1 plus pre-S1+pre-S2 del (0.952)	
		III. pre-S2 plus pre-S1+pre-S2 del (0.093)	
		IV. all three types of pre-S del (1.036)	
12	wild-type	1. **wild-type (97.781)**	1. wild-type (97.781)
		2. pre-S1 del (2.099)	2. pre-S1 del (nt 3026-3205) (1.099)
		3. pre-S2 del (0.112)	3. pre-S2 del (nt 3211-3216) (0.055)
		4. pre-S1+pre-S2 del (0.008)	4. pre-S1+pre-S2 del (nt 3026-3205, 43-138) (0.002)
		I. pre-S1 plus pre-S2 del (2.211)	
		II. pre-S1 plus pre-S1+pre-S2 del (2.107)	
		III. pre-S2 plus pre-S1+pre-S2 del (0.120)	
		IV. all three types of pre-S del (2.219)	
13	wild-type	1. **wild-type (92.291)**	1. wild-type (92.291)
		2. pre-S1 del (4.092)	2. pre-S1 del (nt 2854-2970) (0.650)
		3. pre-S2 del (3.038)	3. pre-S2 del (nt 1-54) (2.641)
		4. pre-S1+pre-S2 del (0.578)	4. pre-S1+pre-S2 del (nt 2855-2872, 1-54) (0.435)
		I. pre-S1 plus pre-S2 del (7.130)	
		II. pre-S1 plus pre-S1+pre-S2 del (4.670)	

		III. pre-S2 plus pre-S1+pre-S2 del (3.616)<br>IV. all three types of pre-S del (7.709)	
14	wild-type	1. **wild-type (69.001)**<br>2. **pre-S1 del (20.530)**<br>3**. pre-S2 del (9.463)**<br>4. pre-S1+pre-S2 del (1.006)<br>I. pre-S1 plus pre-S2 del (29.993)<br>II. pre-S1 plus pre-S1+pre-S2 del (21.536)<br>III. pre-S2 plus pre-S1+pre-S2 del (10.469)<br>IV. all three types of pre-S del (31.999)	1. wild-type (69.001)<br>2. pre-S1 del (nt 3110-3127) (4.779)<br>3. pre-S2 del (nt 1-54) (8.226)<br>4. pre-S1+pre-S2 del (nt 2855-2872, 1-18, 26-59) (0.259)
15	wild-type	1. **wild-type (50.938)**<br>2. **pre-S1 del (19.760)**<br>3. **pre-S1+pre-S2 del (15.021)**<br>4. **pre-S2 del (14.280)**<br>I. pre-S1 plus pre-S2 del (34.040)<br>II. pre-S1 plus pre-S1+pre-S2 del (34.781)<br>III. pre-S2 plus pre-S1+pre-S2 del (29.301)<br>IV. all three types of pre-S del (49.062)	1. wild-type (50.938)<br>2. pre-S1 del (nt 2854-2970) (5.470)<br>3. pre-S1+pre-S2 del (nt 2855-2872, 1-54) (12.421)<br>4. pre-S2 del (nt 1-54) (13.606)
16	wild-type	1. **wild-type (75.230)**<br>2. **pre-S1+pre-S2 del (13.878)**<br>3. **pre-S1 del (10.730)**<br>4. pre-S2 del (0.162)<br>I. pre-S1 plus pre-S2 del (10.892)<br>II. pre-S1 plus pre-S1+pre-S2 del (24.608)<br>III. pre-S2 plus pre-S1+pre-S2 del (14.040)<br>IV. all three types of pre-S del (24.770)	1. wild-type (75.230)<br>2. pre-S1+pre-S2 del (nt 2860-2880, 2954-3097, 1-15) (11.572)<br>3. pre-S1 del (nt 2860-2880, 2954-3097) (9.550)<br>4. pre-S2 del (nt 1-15) (0.138)

17	wild-type	1. **wild-type (98.571)**<br>2. pre-S1 del (1.173)<br>3. pre-S2 del (0.178)<br>4. pre-S1+pre-S2 del (0.078)<br>I. pre-S1 plus pre-S2 del (1.351)<br>II. pre-S1 plus pre-S1+pre-S2 del (1.251)<br>III. pre-S2 plus pre-S1+pre-S2 del (0.256)<br>IV. all three types of pre-S del (1.429)	1. wild-type (98.571)<br>2. pre-S1 del (nt 3106-3129) (0.242)<br>3. pre-S2 del (nt 2-149) (0.043)<br>4. pre-S1+pre-S2 del (nt 2854-2985, 27-149) (0.014)
18	wild-type	1. **wild-type (96.915)**<br>2. pre-S1 del (2.701)<br>3. pre-S2 del (0.364)<br>4. pre-S1+pre-S2 del (0.020)<br>I. pre-S1 plus pre-S2 del (3.065)<br>II. pre-S1 plus pre-S1+pre-S2 del (2.721)<br>III. pre-S2 plus pre-S1+pre-S2 del (0.384)<br>IV. all three types of pre-S del (3.085)	1. wild-type (96.915)<br>2. pre-S1 del (nt 3026-3205) (1.755)<br>3. pre-S2 del (nt 25-54) (0.148)<br>4. pre-S1+pre-S2 del (nt 2854-2988, 45-149) (0.004)
19	wild-type	1. **wild-type (98.069)**<br>2. pre-S1 del (1.263)<br>3. pre-S2 del (0.609)<br>4. pre-S1+pre-S2 del (0.059)<br>I. pre-S1 plus pre-S2 del (1.872)<br>II. pre-S1 plus pre-S1+pre-S2 del (1.322)<br>III. pre-S2 plus pre-S1+pre-S2 del (0.668)<br>IV. all three types of pre-S del (1.931)	1. wild-type (98.069)<br>2. pre-S1 del (nt 3103-3126) (0.253)<br>3. pre-S2 del (nt 1-15) (0.535)<br>4. pre-S1+pre-S2 del (nt 2854-2996, 44-144) (0.015)

20	wild-type	1. **wild-type (69.571)**<br>2. **pre-S2 del (18.590)**<br>**3. pre-S1+pre-S2 del (8.224)**<br>4. pre-S1 del (3.615)<br>I. pre-S1 plus pre-S2 del (22.205)<br>II. pre-S1 plus pre-S1+pre-S2 del (11.839)<br>III. pre-S2 plus pre-S1+pre-S2 del (26.814)<br>IV. all three types of pre-S del (30.429)	1. wild-type (69.571)<br>2. pre-S2 del (nt 1-54) (18.482)<br>3. pre-S1+pre-S2 del (nt 2855-2872, 1-54) (7.712)<br>4. pre-S1 del (nt 2855-2872) (2.674)
21	wild-type	1. **pre-S2 del (41.477)**<br>2. **pre-S1+pre-S2 del (39.126)**<br>3. **wild-type (12.348)**<br>4. **pre-S1 del (7.048)**<br>I. pre-S1 plus pre-S2 del (48.525)<br>II. pre-S1 plus pre-S1+pre-S2 del (46.174)<br>III. pre-S2 plus pre-S1+pre-S2 del (80.603)<br>IV. all three types of pre-S del (87.652)	1. pre-S2 del (nt 1-54) (24.620)<br>2. pre-S1+pre-S2 del (nt 2855-2872, 1-54) (26.451)<br>3. wild-type (12.348)<br>4. pre-S1 del (nt 2855-2872) (4.978)
22	1. wild-type<br>2. pre-S1 del (nt 2855-2872)	1. **pre-S2 del (42.909)**<br>2. **pre-S1+pre-S2 del (27.915)**<br>3. **wild-type (17.564)**<br>4. **pre-S1 del (11.612)**<br>I. pre-S1 plus pre-S2 del (54.521)<br>II. pre-S1 plus pre-S1+pre-S2 del (39.527)<br>III. pre-S2 plus pre-S1+pre-S2 del (70.824)<br>IV. all three types of pre-S del (82.436)	1. pre-S2 del (nt 1-54) (38.091)<br>2. pre-S1+pre-S2 del (2855-2872, 1-54) (25.090)<br>3. wild-type (17.564)<br>4. pre-S1 del (nt 2855-2872) (8.072)

23	1. wild-type<br>2. pre-S1+pre-S2 del<br>(nt 2855-2872, 1-54)	1. **wild-type (52.661)**<br>2. **pre-S2 del (25.093)**<br>3. **pre-S1+pre-S2 del (11.424)**<br>4. **pre-S1 del (10.821)**<br>I. pre-S1 plus pre-S2 del (36.724)<br>II. pre-S1 plus pre-S1+pre-S2 del (22.245)<br>III. pre-S2 plus pre-S1+pre-S2 del (36.517)<br>IV. all three types of pre-S del (47.339)	1. wild-type (52.661)<br>2. pre-S2 del (nt 1-54) (18.682)<br>3. pre-S1+pre-S2 del (nt 2855-2872, 1-54) (8.344)<br>4. pre-S1 del (nt 2855-2872) (2.817)
24	wild-type	1. **wild-type (96.073)**<br>2. pre-S1 del (1.895)<br>3. pre-S1+pre-S2 del (1.202)<br>4. pre-S2 del (0.830)<br>I. pre-S1 plus pre-S2 del (2.725)<br>II. pre-S1 plus pre-S1+pre-S2 del (3.097)<br>III. pre-S2 plus pre-S1+pre-S2 del (2.032)<br>IV. all three types of pre-S del (3.927)	1. wild-type (96.073)<br>2. pre-S1 del (nt 2954-3097) (0.632)<br>3. pre-S1+pre-S2 del (nt 2855-2872, 1-54) (0.452)<br>4. pre-S2 del (nt 1-54) (0.382)
25	wild-type	1. **wild-type (93.547)**<br>2. pre-S1 del (4.345)<br>3. pre-S2 del (1.844)<br>4. pre-S1+pre-S2 del (0.265)<br>I. pre-S1 plus pre-S2 del (6.189)<br>II. pre-S1 plus pre-S1+pre-S2 del (4.610)<br>III. pre-S2 plus pre-S1+pre-S2 del (2.109)<br>IV. all three types of pre-S del (6.453)	1. wild-type (93.547)<br>2. pre-S1 del (nt 3026-3205) (0.869)<br>3. pre-S2 del (nt 1-54) (1.107)<br>4. pre-S1+pre-S2 del (nt 2855-2872, 2897-2923, 1-54) (0.180)

26	wild-type	1. **wild-type (97.921)**<br>2. pre-S1 del (1.388)<br>3. pre-S2 del (0.691)<br>4. pre-S1+pre-S2 del (0.000)<br>I. pre-S1 plus pre-S2 del (2.079)<br>II. pre-S1 plus pre-S1+pre-S2 del (1.388)<br>III. pre-S2 plus pre-S1+pre-S2 del (0.691)<br>IV. all three types of pre-S del (2.079)	1. wild-type (97.921)<br>2. pre-S1 del (nt 3067-3162) (0.040)<br>3. pre-S2 del (nt 3211-3216) (0.046)<br>4. pre-S1+pre-S2 del (0.000)
27	pre-S1+pre-S2 del<br>(nt 2855-2872, 1-54)	1. **wild-type (60.195)**<br>2. **pre-S2 del (22.882)**<br>3. **pre-S1+pre-S2 del (9.280)**<br>4. **pre-S1 del (7.643)**<br>I. pre-S1 plus pre-S2 del (30.525)<br>II. pre-S1 plus pre-S1+pre-S2 del (16.923)<br>III. pre-S2 plus pre-S1+pre-S2 del (32.162)<br>IV. all three types of pre-S del (39.805)	1. wild-type (60.195)<br>2. pre-S2 del (nt 1-57) (22.473)<br>3. pre-S1+pre-S2 del (nt 2855-2872, 1-54) (4.815)<br>4. pre-S1 del (nt 3025-3126) (4.396)
28	wild-type	1. **pre-S1 del (75.113)**<br>2. **wild-type (11.506)**<br>3. **pre-S1+pre-S2 del (10.472)**<br>4. pre-S2 del (2.909)<br>I. pre-S1 plus pre-S2 del (78.022)<br>II. pre-S1 plus pre-S1+pre-S2 del (85.585)<br>III. pre-S2 plus pre-S1+pre-S2 del (13.381)<br>IV. all three types of pre-S del (88.494)	1. pre-S1 del (nt 2855-2872) (5.979)<br>2. wild-type (11.506)<br>3. pre-S1+pre-S2 del (nt 2855-2872, 1-54) (8.016)<br>4. pre-S2 del (nt 1-54) (2.747)

29	wild-type	1. **wild-type (97.730)**<br>2. pre-S1 del (1.691)<br>3. pre-S2 del (0.575)<br>4. pre-S1+pre-S2 del (0.004)<br>I. pre-S1 plus pre-S2 del (2.266)<br>II. pre-S1 plus pre-S1+pre-S2 del (1.695)<br>III. pre-S2 plus pre-S1+pre-S2 del (0.579)<br>IV. all three types of pre-S del (2.270)	1. wild-type (97.730)<br>2. pre-S1 del (nt 2855-2970) (0.217)<br>3. pre-S2 del (nt 1-12) (0.323)<br>4. pre-S1+pre-S2 del (nt 2855-2970, 1-54) (0.004)
30	pre-S1 del (nt 3010-3075)	1. **wild-type (87.018)**<br>2. **pre-S1 del (11.703)**<br>3. pre-S2 del (1.113)<br>4. pre-S1+pre-S2 del (0.166)<br>I. pre-S1 plus pre-S2 del (12.816)<br>II. pre-S1 plus pre-S1+pre-S2 del (11.869)<br>III. pre-S2 plus pre-S1+pre-S2 del (1.279)<br>IV. all three types of pre-S del (12.982)	1. wild-type (87.018)<br>2. pre-S1 del (nt 3010-3075) (5.189)<br>3. pre-S2 del (nt 1-9) (0.392)<br>4. pre-S1+pre-S2 del (nt 3010-3075, 1-9) (0.046)
31	pre-S1 del (nt 2910-3089)	1. **wild-type (78.709)**<br>2. **pre-S1 del (20.616)**<br>3. pre-S2 del (0.646)<br>4. pre-S1+pre-S2 del (0.029)<br>I. pre-S1 plus pre-S2 del (21.262)<br>II. pre-S1 plus pre-S1+pre-S2 del (20.645)<br>III. pre-S2 plus pre-S1+pre-S2 del (0.675)<br>IV. all three types of pre-S del (21.291)	1. wild-type (78.709)<br>2. pre-S1 del (nt 2910-3089) (9.181)<br>3. pre-S2 del (nt 1-15) (0.345)<br>4. pre-S1+pre-S2 del (nt 2910-3055, 3067-3089, 4-12) (0.007)

32	wild-type	1. **wild-type (98.105)**<br>2. pre-S1 del (1.152)<br>3. pre-S2 del (0.733)<br>4. pre-S1+pre-S2 del (0.010)<br>I. pre-S1 plus pre-S2 del (1.885)<br>II. pre-S1 plus pre-S1+pre-S2 del (1.162)<br>III. pre-S2 plus pre-S1+pre-S2 del (0.743)<br>IV. all three types of pre-S del (1.895)	1. wild-type (98.105)<br>2. pre-S1 del (nt 2855-2970) (0.060)<br>3. pre-S2 del (nt 1-9) (0.408)<br>4. pre-S1+pre-S2 del (nt 2855-2872, 1-54) (0.003)
33	wild-type	1. **wild-type (80.067)**<br>2. **pre-S2 del (9.477)**<br>3. **pre-S1 del (8.993)**<br>4. pre-S1+pre-S2 del (1.463)<br>I. pre-S1 plus pre-S2 del (18.470)<br>II. pre-S1 plus pre-S1+pre-S2 del (10.456)<br>III. pre-S2 plus pre-S1+pre-S2 del (10.940)<br>IV. all three types of pre-S del (19.933)	1. wild-type (80.067)<br>2. pre-S2 del (nt 1-12) (8.957)<br>3. pre-S1 del (nt 2866-3075) (7.322)<br>4. pre-S1+pre-S2 del (nt 2866-3075, 1-9) (0.871)
34	wild-type	1. **wild-type (80.175)**<br>2. **pre-S2 del (13.639)**<br>3. **pre-S1 del (5.169)**<br>4. pre-S1+pre-S2 del (1.017)<br>I. pre-S1 plus pre-S2 del (18.808)<br>II. pre-S1 plus pre-S1+pre-S2 del (6.186)<br>III. pre-S2 plus pre-S1+pre-S2 del (14.656)<br>IV. all three types of pre-S del (19.825)	1. wild-type (80.175)<br>2. pre-S2 del (nt 1-30) (4.394)<br>3. pre-S1 del (nt 2865-2975) (2.356)<br>4. pre-S1+pre-S2 del (nt 2866-2975, 1-29) (0.212)

35	wild-type	1. **wild-type (92.156)**<br>2. **pre-S1 del (7.022)**<br>3. pre-S2 del (0.793)<br>4. pre-S1+pre-S2 del (0.029)<br>I. pre-S1 plus pre-S2 del (7.815)<br>II. pre-S1 plus pre-S1+pre-S2 del (7.051)<br>III. pre-S2 plus pre-S1+pre-S2 del (0.822)<br>IV. all three types of pre-S del (7.844)	1. wild-type (92.156)<br>2. pre-S1 del (nt 2854-3018) (1.346)<br>3. pre-S2 del (nt 1-9) (0.323)<br>4. pre-S1+pre-S2 del (nt 2855-2872, 1-9) (0.004)
36	1. pre-S1 del (nt 2854-2970)<br>2. pre-S1+pre-S2 del (nt 2855-2872, 1-54)	1. **pre-S1+pre-S2 del (40.433)**<br>2. **pre-S1 del (34.174)**<br>3. **wild-type (20.822)**<br>4. pre-S2 del (4.571)<br>I. pre-S1 plus pre-S2 del (38.745)<br>II. pre-S1 plus pre-S1+pre-S2 del (74.607)<br>III. pre-S2 plus pre-S1+pre-S2 del (45.004)<br>IV. all three types of pre-S del (79.178)	1. pre-S1+pre-S2 del (nt 2855-2872, 1-54) (22.713)<br>2. pre-S1 del (nt 2854-2970) (22.956)<br>3. wild-type (20.822)<br>4. pre-S2 del (nt 1-54) (4.380)
37	wild-type	1. **wild-type (80.758)**<br>2. **pre-S2 del (12.910)**<br>3. **pre-S1 del (5.629)**<br>4. pre-S1+pre-S2 del (0.704)<br>I. pre-S1 plus pre-S2 del (18.539)<br>II. pre-S1 plus pre-S1+pre-S2 del (6.333)<br>III. pre-S2 plus pre-S1+pre-S2 del (13.614)<br>IV. all three types of pre-S del (19.242)	1. wild-type (80.758)<br>2. pre-S2 del (nt 1-54) (6.944)<br>3. pre-S1 del (nt 2855-2875) (4.209)<br>4. pre-S1+pre-S2 del (nt 2855-2875, 1-54) (0.253)

38	pre-S1 del (nt 3021-3203)	1. **wild-type (61.320)**<br>2. **pre-S1 del (34.045)**<br>3. pre-S2 del (3.491)<br>4. pre-S1+pre-S2 del (1.144)<br>I. pre-S1 plus pre-S2 del (37.536)<br>II. pre-S1 plus pre-S1+pre-S2 del (35.189)<br>III. pre-S2 plus pre-S1+pre-S2 del (4.635)<br>IV. all three types of pre-S del (38.680)	1. wild-type (61.320)<br>2. pre-S1 del (nt 3021-3203) (23.818)<br>3. pre-S2 del (nt 6-41) (1.385)<br>4. pre-S1+pre-S2 del (nt 2855-2872, 2919-3126, 6-41) (0.163)
39	wild-type	1. **wild-type (97.172)**<br>2. pre-S1 del (1.957)<br>3. pre-S2 del (0.850)<br>4. pre-S1+pre-S2 del (0.021)<br>I. pre-S1 plus pre-S2 del (2.807)<br>II. pre-S1 plus pre-S1+pre-S2 del (1.978)<br>III. pre-S2 plus pre-S1+pre-S2 del (0.871)<br>IV. all three types of pre-S del (2.828)	1. wild-type (97.172)<br>2. pre-S1 del (nt 3021-3203) (0.353)<br>3. pre-S2 del (nt 1-12) (0.488)<br>4. pre-S1+pre-S2 del (nt 3078-3094, 6-41) (0.003)
40	wild-type	1. **wild-type (97.501)**<br>2. pre-S1 del (1.586)<br>3. pre-S2 del (0.908)<br>4. pre-S1+pre-S2 del (0.005)<br>I. pre-S1 plus pre-S2 del (2.494)<br>II. pre-S1 plus pre-S1+pre-S2 del (1.591)<br>III. pre-S2 plus pre-S1+pre-S2 del (0.913)<br>IV. all three types of pre-S del (2.499)	1. wild-type (97.501)<br>2. pre-S1 del (nt 3107-3202) (0.081)<br>3. pre-S2 del (nt 1-12) (0.529)<br>4. pre-S1+pre-S2 del (nt 3133-3136, 1-10) (0.005)

41	pre-S1 del (nt 2858-2986)	1. **pre-S1 del (84.262)**<br>2. **wild-type (14.798)**<br>3. pre-S1+pre-S2 del (0.779)<br>4. pre-S2 del (0.161)<br>I. pre-S1 plus pre-S2 del (84.423)<br>II. pre-S1 plus pre-S1+pre-S2 del (85.041)<br>III. pre-S2 plus pre-S1+pre-S2 del (0.940)<br>IV. all three types of pre-S del (85.202)	1. pre-S1 del (nt 2858-2986) (82.700)<br>2. wild-type (14.798)<br>3. pre-S1+pre-S2 del (nt 2858-2981, 1-10) (0.268)<br>4. pre-S2 del (nt 1-33) (0.053)
42	wild-type	1. **wild-type (97.757)**<br>2. pre-S1 del (1.305)<br>3. pre-S2 del (0.923)<br>4. pre-S1+pre-S2 del (0.015)<br>I. pre-S1 plus pre-S2 del (2.228)<br>II. pre-S1 plus pre-S1+pre-S2 del (1.320)<br>III. pre-S2 plus pre-S1+pre-S2 del (0.938)<br>IV. all three types of pre-S del (2.243)	1. wild-type (97.757)<br>2. pre-S1 del (nt 3106-3207) (0.060)<br>3. pre-S2 del (nt 3211-3213) (0.124)<br>4. pre-S1+pre-S2 del (nt 3069-3118, 3211-3212) (0.004)
43	wild-type	1. **pre-S2 del (49.695)**<br>2. **wild-type (49.248)**<br>3. pre-S1+pre-S2 del (0.814)<br>4. pre-S1 del (0.242)<br>I. pre-S1 plus pre-S2 del (49.937)<br>II. pre-S1 plus pre-S1+pre-S2 del (1.056)<br>III. pre-S2 plus pre-S1+pre-S2 del (50.509)<br>IV. all three types of pre-S del (50.752)	1. pre-S2 del (nt 37-54) (43.929)<br>2. wild-type (49.248)<br>3. pre-S1+pre-S2 del (nt 3139-3142, 37-54) (0.049)<br>4. pre-S1 del (nt 3103-3200) (0.019)

44	wild-type	1. **wild-type (98.244)**<br>2. pre-S1 del (1.113)<br>3. pre-S2 del (0.643)<br>4. pre-S1+pre-S2 del (0.000)<br>I. pre-S1 plus pre-S2 del (1.756)<br>II. pre-S1 plus pre-S1+pre-S2 del (1.113)<br>III. pre-S2 plus pre-S1+pre-S2 del (0.643)<br>IV. all three types of pre-S del (1.756)	1. wild-type (98.244)<br>2. pre-S1 del (nt 3089-3202) (0.049)<br>3. pre-S2 del (nt 1-15) (0.319)<br>4. pre-S1+pre-S2 del (0.000)
45	wild-type	1. **wild-type (97.534)**<br>2. pre-S1 del (1.376)<br>3. pre-S2 del (1.076)<br>4. pre-S1+pre-S2 del (0.014)<br>I. pre-S1 plus pre-S2 del (2.452)<br>II. pre-S1 plus pre-S1+pre-S2 del (1.390)<br>III. pre-S2 plus pre-S1+pre-S2 del (1.090)<br>IV. all three types of pre-S del (2.466)	1. wild-type (97.534)<br>2. pre-S1 del (nt 3106-3207) (0.063)<br>3. pre-S2 del (nt 1-9) (0.769)<br>4. pre-S1+pre-S2 del (nt 3095-3150, 1-12) (0.007)
46	wild-type	1. **wild-type (97.735)**<br>2. pre-S1 del (1.534)<br>3. pre-S2 del (0.731)<br>4. pre-S1+pre-S2 del (0.000)<br>I. pre-S1 plus pre-S2 del (2.265)<br>II. pre-S1 plus pre-S1+pre-S2 del (1.534)<br>III. pre-S2 plus pre-S1+pre-S2 del (0.731)<br>IV. all three types of pre-S del (2.265)	1. wild-type (97.735)<br>2. pre-S1 del (nt 3138-3197) (0.111)<br>3. pre-S2 del (nt 1-9) (0.371)<br>4. pre-S1+pre-S2 del (0.000)

47	wild-type	1. **wild-type (78.334)**<br>2. **pre-S1 del (20.695)**<br>3. pre-S2 del (0.866)<br>4. pre-S1+pre-S2 del (0.105)<br>I. pre-S1 plus pre-S2 del (21.561)<br>II. pre-S1 plus pre-S1+pre-S2 del (20.800)<br>III. pre-S2 plus pre-S1+pre-S2 del (0.971)<br>IV. all three types of pre-S del (21.666)	1. wild-type (78.334)<br>2. pre-S1 del (nt 2895-3140) (15.887)<br>3. pre-S2 del (nt 1-9) (0.279)<br>4. pre-S1+pre-S2 del (nt 2895-3141, 1-19, 23-27) (0.023)
48	1. pre-S1 del (nt 2968-3093)<br>2. pre-S2 del (nt 1-54)	1. **pre-S1 del (57.159)**<br>2. **wild-type (20.456)**<br>3. **pre-S2 del (13.666)**<br>4. **pre-S1+pre-S2 del (8.719)**<br>I. pre-S1 plus pre-S2 del (70.825)<br>II. pre-S1 plus pre-S1+pre-S2 del (65.878)<br>III. pre-S2 plus pre-S1+pre-S2 del (22.385)<br>IV. all three types of pre-S del (79.544)	1. pre-S1 del (nt 2968-3093) (47.067)<br>2. wild-type (20.456)<br>3. pre-S2 del (nt 1-54) (8.120)<br>4. pre-S1+pre-S2 del (nt 2968-3093, 1-54) (3.469)
49	wild-type	1. **wild-type (75.021)**<br>2. **pre-S2 del (21.983)**<br>3. pre-S1 del (2.327)<br>4. pre-S1+pre-S2 del (0.669)<br>I. pre-S1 plus pre-S2 del (24.310)<br>II. pre-S1 plus pre-S1+pre-S2 del (2.996)<br>III. pre-S2 plus pre-S1+pre-S2 del (22.652)<br>IV. all three types of pre-S del (24.979)	1. wild-type (75.021)<br>2. pre-S2 del (nt 1-1) (19.454)<br>3. pre-S1 del (nt 2855-2872) (0.704)<br>4. pre-S1+pre-S2 del (nt 2855-2872, 1-54) (0.606)

50	wild-type	1. **wild-type (98.169)**<br>2. pre-S1 del (1.138)<br>3. pre-S2 del (0.693)<br>4. pre-S1+pre-S2 del (0.000)<br>I. pre-S1 plus pre-S2 del (1.831)<br>II. pre-S1 plus pre-S1+pre-S2 del (1.138)<br>III. pre-S2 plus pre-S1+pre-S2 del (0.693)<br>IV. all three types of pre-S del (1.831)	1. wild-type (98.169)<br>2. pre-S1 del (nt 3138-3197) (0.067)<br>3. pre-S2 del (nt 1-9) (0.377)<br>4. pre-S1+pre-S2 del (0.000)
51	wild-type	1. **wild-type (97.734)**<br>2. pre-S1 del (1.546)<br>3. pre-S2 del (0.719)<br>4. pre-S1+pre-S2 del (0.000)<br>I. pre-S1 plus pre-S2 del (2.265)<br>II. pre-S1 plus pre-S1+pre-S2 del (1.546)<br>III. pre-S2 plus pre-S1+pre-S2 del (0.719)<br>IV. all three types of pre-S del (2.266)	1. wild-type (97.734)<br>2. pre-S1 del (nt 3104-3202) (0.165)<br>3. pre-S2 del (nt 1-9) (0.377)<br>4. pre-S1+pre-S2 del (0.000)
52	wild-type	1. **wild-type (98.167)**<br>2. pre-S1 del (1.192)<br>3. pre-S2 del (0.617)<br>4. pre-S1+pre-S2 del (0.024)<br>I. pre-S1 plus pre-S2 del (1.809)<br>II. pre-S1 plus pre-S1+pre-S2 del (1.216)<br>III. pre-S2 plus pre-S1+pre-S2 del (0.641)<br>IV. all three types of pre-S del (1.833)	1. wild-type (98.167)<br>2. pre-S1 del (nt 2854-3128) (0.132)<br>3. pre-S2 del (nt 1-9) (0.246)<br>4. pre-S1+pre-S2 del (nt 3022-3125, 1-55) (0.018)

53	wild-type	1. **wild-type (97.002)**<br>2. pre-S1 del (2.144)<br>3. pre-S2 del (0.849)<br>4. pre-S1+pre-S2 del (0.005)<br>I. pre-S1 plus pre-S2 del (2.993)<br>II. pre-S1 plus pre-S1+pre-S2 del (2.149)<br>III. pre-S2 plus pre-S1+pre-S2 del (0.854)<br>IV. all three types of pre-S del (2.998)	1. wild-type (97.002)<br>2. pre-S1 del (nt 3026-3205) (0.927)<br>3. pre-S2 del (nt 1-9) (0.528)<br>4. pre-S1+pre-S2 del (nt 3020-3119, 1-55) (0.005)
54	wild-type	1. **wild-type (96.687)**<br>2. pre-S1 del (2.625)<br>3. pre-S2 del (0.676)<br>4. pre-S1+pre-S2 del (0.013)<br>I. pre-S1 plus pre-S2 del (3.301)<br>II. pre-S1 plus pre-S1+pre-S2 del (2.638)<br>III. pre-S2 plus pre-S1+pre-S2 del (0.689)<br>IV. all three types of pre-S del (3.313)	1. wild-type (96.687)<br>2. pre-S1 del (nt 2855-2872) (1.117)<br>3. pre-S2 del (nt 1-12) (0.411)<br>4. pre-S1+pre-S2 del (nt 2855-2872, 1-17) (0.004)
55	wild-type	1. **wild-type (64.666)**<br>2. **pre-S1 del (34.582)**<br>3. pre-S2 del (0.493)<br>4. pre-S1+pre-S2 del (0.259)<br>I. pre-S1 plus pre-S2 del (35.075)<br>II. pre-S1 plus pre-S1+pre-S2 del (34.841)<br>III. pre-S2 plus pre-S1+pre-S2 del (0.752)<br>IV. all three types of pre-S del (35.334)	1. wild-type (64.666)<br>2. pre-S1 del (nt 2858-2986) (33.466)<br>3. pre-S2 del (nt 1-9) (0.303)<br>4. pre-S1+pre-S2 del (nt 2858-2981, 1-10) (0.069)

56	wild-type	1. **pre-S1 del (69.372)**<br>2. **wild-type (30.404)**<br>3. pre-S2 del (0.218)<br>4. pre-S1+pre-S2 del (0.005)<br>I. pre-S1 plus pre-S2 del (69.590)<br>II. pre-S1 plus pre-S1+pre-S2 del (69.377)<br>III. pre-S2 plus pre-S1+pre-S2 del (0.223)<br>IV. all three types of pre-S del (69.596)	1. pre-S1 del (nt 2856-3101) (66.854)<br>2. wild-type (30.404)<br>3. pre-S2 del (nt 1-12) (0.130)<br>4. pre-S1+pre-S2 del (nt 2984-3098, 1-10) (0.002)
57	wild-type	1. **wild-type (94.005)**<br>2. pre-S1 del (3.729)<br>3. pre-S2 del (1.824)<br>4. pre-S1+pre-S2 del (0.442)<br>I. pre-S1 plus pre-S2 del (5.553)<br>II. pre-S1 plus pre-S1+pre-S2 del (4.171)<br>III. pre-S2 plus pre-S1+pre-S2 del (2.266)<br>IV. all three types of pre-S del (5.995)	1. wild-type (94.005)<br>2. pre-S1 del (nt 2895-3188) (0.677)<br>3. pre-S2 del (nt 1-54) (1.029)<br>4. pre-S1+pre-S2 del (nt 2855-2872, 1-54) (0.253)
58	wild-type	1. **wild-type (95.575)**<br>2. pre-S1 del (2.481)<br>3. pre-S2 del (1.931)<br>4. pre-S1+pre-S2 del (0.013)<br>I. pre-S1 plus pre-S2 del (4.412)<br>II. pre-S1 plus pre-S1+pre-S2 del (2.494)<br>III. pre-S2 plus pre-S1+pre-S2 del (1.944)<br>IV. all three types of pre-S del (4.424)	1. wild-type (95.575)<br>2. pre-S1 del (nt 3039-3092) (0.590)<br>3. pre-S2 del (nt 1-12) (0.510)<br>4. pre-S1+pre-S2 del (nt 2854-2940, 1-144) (0.007)

59	pre-S2 del (nt 15-56)	1. **pre-S2 del (64.182)**<br>2. **wild-type (32.367)**<br>3. pre-S1+pre-S2 del (2.975)<br>4. pre-S1 del (0.476)<br>I. pre-S1 plus pre-S2 del (64.658)<br>II. pre-S1 plus pre-S1+pre-S2 del (3.451)<br>III. pre-S2 plus pre-S1+pre-S2 del (67.157)<br>IV. all three types of pre-S del (67.633)	1. pre-S2 del (nt 15-56) (33.613)<br>2. wild-type (32.367)<br>3. pre-S1+pre-S2 del (nt 3088-3126, 15-56) (0.176)<br>4. pre-S1 del (nt 2923-3090) (0.073)
60	wild-type	1. **pre-S1 del (38.192)**<br>2. **wild-type (34.644)**<br>3. **pre-S2 del (19.195)**<br>4. **pre-S1+pre-S2 del (7.968)**<br>I. pre-S1 plus pre-S2 del (57.387)<br>II. pre-S1 plus pre-S1+pre-S2 del (46.160)<br>III. pre-S2 plus pre-S1+pre-S2 del (27.163)<br>IV. all three types of pre-S del (65.356)	1. pre-S1 del (nt 2854-2970) (27.998)<br>2. wild-type (30.404)<br>3. pre-S2 del (nt 1-54) (18.373)<br>4. pre-S1+pre-S2 del (nt 3025-3126, 1-57) (2.383)
61	pre-S1 del (nt 2855-2965)	1. **pre-S1 del (40.086)**<br>2. **wild-type (34.919)**<br>3. **pre-S2 del (16.065)**<br>4. **pre-S1+pre-S2 del (8.930)**<br>I. pre-S1 plus pre-S2 del (56.151)<br>II. pre-S1 plus pre-S1+pre-S2 del (49.016)<br>III. pre-S2 plus pre-S1+pre-S2 del (24.995)<br>IV. all three types of pre-S del (65.081)	1. pre-S1 del (nt 2855-2965) (24.125)<br>2. wild-type (34.919)<br>3. pre-S2 del (nt 1-54) (15.565)<br>4. pre-S1+pre-S2 del (nt 3022-3126, 1-60) (2.911)

62	pre-S1+pre-S2 del<br>(nt 2855-2872, 1-54)	1. **pre-S1 del (29.181)**<br>2. **wild-type (23.836)**<br>3. **pre-S2 del (23.645)**<br>4. **pre-S1+pre-S2 del (23.338)**<br>I. pre-S1 plus pre-S2 del (52.826)<br>II. pre-S1 plus pre-S1+pre-S2 del (52.519)<br>III. pre-S2 plus pre-S1+pre-S2 del (46.983)<br>IV. all three types of pre-S del (76.164)	1. pre-S1 del (nt 2856-2969) (17.015)<br>2. wild-type (23.836)<br>3. pre-S2 del (nt 1-54) (23.402)<br>4. pre-S1+pre-S2 del (nt 2855-2872, 1-54) (15.956)
63	pre-S1 del (nt 2854-2970)	1. **pre-S1 del (52.401)**<br>2. **wild-type (42.979)**<br>3. pre-S2 del (2.312)<br>4. pre-S1+pre-S2 del (2.308)<br>I. pre-S1 plus pre-S2 del (54.713)<br>II. pre-S1 plus pre-S1+pre-S2 del (54.709)<br>III. pre-S2 plus pre-S1+pre-S2 del (4.620)<br>IV. all three types of pre-S del (57.021)	1. pre-S1 del (nt 2854-2970) (43.037)<br>2. wild-type (42.979)<br>3. pre-S2 del (nt 1-54) (1.977)<br>4. pre-S1+pre-S2 del (nt 2860-2880, 2954-3097, 1-15) (1.445)
64	wild-type	1. **pre-S1 del (41.155)**<br>2. **wild-type (37.573)**<br>3. **pre-S2 del (18.905)**<br>4. pre-S1+pre-S2 del (2.367)<br>I. pre-S1 plus pre-S2 del (60.060)<br>II. pre-S1 plus pre-S1+pre-S2 del (43.522)<br>III. pre-S2 plus pre-S1+pre-S2 del (21.272)<br>IV. all three types of pre-S del (62.427)	1. pre-S1 del (nt 2854-2970) (35.773)<br>2. wild-type (37.573)<br>3. pre-S2 del (nt 1-54) (18.494)<br>4. pre-S1+pre-S2 del (nt 2855-2970, 1-54) (1.676)

65	wild-type	1. **pre-S1 del (63.334)**<br>2. **wild-type (32.806)**<br>3. pre-S2 del (1.965)<br>4. pre-S1+pre-S2 del (1.895)<br>I. pre-S1 plus pre-S2 del (65.229)<br>II. pre-S1 plus pre-S1+pre-S2 del (65.229)<br>III. pre-S2 plus pre-S1+pre-S2 del (3.860)<br>IV. all three types of pre-S del (67.194)	1. pre-S1 del (nt 2854-2970) (33.450)<br>2. wild-type (32.806)<br>3. pre-S2 del (nt 1-54) (1.708)<br>4. pre-S1+pre-S2 del (nt 2855-2970, 1-54) (0.994)
66	pre-S1+pre-S2 del<br>(nt 2855-2872, 1-54)	1. **wild-type (30.973)**<br>2. **pre-S1+pre-S2 del (27.774)**<br>3. **pre-S1 del (27.161)**<br>4. **pre-S2 del (14.091)**<br>I. pre-S1 plus pre-S2 del (41.252)<br>II. pre-S1 plus pre-S1+pre-S2 del (54.935)<br>III. pre-S2 plus pre-S1+pre-S2 del (41.865)<br>IV. all three types of pre-S del (69.027)	1. wild-type (30.973)<br>2. pre-S1+pre-S2 del (nt 2855-2872, 1-54) (24.398)<br>3. pre-S1 del (nt 2854-2970) (14.813)<br>4. pre-S2 del (nt 1-54) (13.846)
67	wild-type	1. **wild-type (46.914)**<br>2. **pre-S1 del (45.517)**<br>3. **pre-S2 del (6.834)**<br>4. pre-S1+pre-S2 del (0.735)<br>I. pre-S1 plus pre-S2 del (52.351)<br>II. pre-S1 plus pre-S1+pre-S2 del (46.252)<br>III. pre-S2 plus pre-S1+pre-S2 del (7.569)<br>IV. all three types of pre-S del (53.086)	1. wild-type (46.914)<br>2. pre-S1 del (nt 2854-2970) (37.666)<br>3. pre-S2 del (nt 1-54) (5.216)<br>4. pre-S1+pre-S2 del (nt 2854-2970, 1-54) (0.307)

68	pre-S1+pre-S2 del<br>(nt 2855-2872, 1-54)	1. **pre-S1 del (43.130)**<br>2. **wild-type (41.965)**<br>3. **pre-S2 del (9.508)**<br>4. **pre-S1+pre-S2 del (5.397)**<br>I. pre-S1 plus pre-S2 del (52.638)<br>II. pre-S1 plus pre-S1+pre-S2 del (48.527)<br>III. pre-S2 plus pre-S1+pre-S2 del (14.905)<br>IV. all three types of pre-S del (58.035)	1. pre-S1 del (nt 2854-2970) (35.888)<br>2. wild-type (41.965)<br>3. pre-S2 del (nt 1-54) (9.239)<br>4. pre-S1+pre-S2 del (nt 2855-2872, 1-54) (4.160)
69	pre-S1+pre-S2 del<br>(nt 2855-2872, 1-54)	1. **wild-type (36.868)**<br>2. **pre-S1 del (35.238)**<br>3. **pre-S2 del (21.130)**<br>4. **pre-S1+pre-S2 del (6.763)**<br>I. pre-S1 plus pre-S2 del (56.368)<br>II. pre-S1 plus pre-S1+pre-S2 del (42.001)<br>III. pre-S2 plus pre-S1+pre-S2 del (27.893)<br>IV. all three types of pre-S del (63.132)	1. wild-type (36.868)<br>2. pre-S1 del (nt 2854-2970) (29.499)<br>3. pre-S2 del (nt 1-54) (20.218)<br>4. pre-S1+pre-S2 del (nt 2855-2872, 1-54) (3.332)
70	pre-S1 del (nt 2854-3021)	1. **wild-type (94.788)**<br>2. **pre-S1 del (4.643)**<br>3. pre-S2 del (0.455)<br>4. pre-S1+pre-S2 del (0.114)<br>I. pre-S1 plus pre-S2 del (5.098)<br>II. pre-S1 plus pre-S1+pre-S2 del (4.757)<br>III. pre-S2 plus pre-S1+pre-S2 del (0.569)<br>IV. all three types of pre-S del (5.212)	1. wild-type (94.788)<br>2. pre-S1 del (nt 2854-3021) (3.404)<br>3. pre-S2 del (nt 1-15) (0.192)<br>4. pre-S1+pre-S2 del (nt 2855-2872, 1-54) (0.086)

71	wild-type	1. **pre-S2 del (54.981)**<br>2. **wild-type (42.238)**<br>3. pre-S1+pre-S2 del (2.274)<br>4. pre-S1 del (0.507)<br>I. pre-S1 plus pre-S2 del (55.488)<br>II. pre-S1 plus pre-S1+pre-S2 del (2.781)<br>III. pre-S2 plus pre-S1+pre-S2 del (57.255)<br>IV. all three types of pre-S del (57.762)	1. pre-S2 del (nt 48-56) (52.982)<br>2. wild-type (42.238)<br>3. pre-S1+pre-S2 del (nt 3026-3205, 48-56) (0.916)<br>4. pre-S1 del (nt 2854-3021) (0.334)
72	pre-S1 del (nt 2854-3021)	1. **wild-type (91.511)**<br>2. **pre-S1 del (7.310)**<br>3. pre-S2 del (0.807)<br>4. pre-S1+pre-S2 del (0.373)<br>I. pre-S1 plus pre-S2 del (8.117)<br>II. pre-S1 plus pre-S1+pre-S2 del (7.683)<br>III. pre-S2 plus pre-S1+pre-S2 del (1.180)<br>IV. all three types of pre-S del (8.489)	1. wild-type (91.511)<br>2. pre-S1 del (nt 2854-3021) (5.744)<br>3. pre-S2 del (nt 48-56) (0.294)<br>4. pre-S1+pre-S2 del (nt 2855-2872, 1-54) (0.322)
73	pre-S1+pre-S2 del (nt 2855-2872, 3012-3086, 1-51)	1. **wild-type (52.297)**<br>2. **pre-S1+pre-S2 del (31.339)**<br>3. **pre-S1 del (12.863)**<br>4. pre-S2 del (3.502)<br>I. pre-S1 plus pre-S2 del (16.365)<br>II. pre-S1 plus pre-S1+pre-S2 del (44.202)<br>III. pre-S2 plus pre-S1+pre-S2 del (34.841)<br>IV. all three types of pre-S del (47.703)	1. wild-type (52.297)<br>2. pre-S1+pre-S2 del (nt 2855-2872, 3012-3086, 1-51) (8.935)<br>3. pre-S1 del (nt 2855-2872, 3012-3092) (6.480)<br>4. pre-S2 del (nt 1-51) (2.094)

74	pre-S2 del (nt 1-57)	1. **wild-type (58.984)**	1. wild-type (58.984)
		2. **pre-S2 del (34.533)**	2. pre-S2 del (nt 1-57) (20.926)
		3. pre-S1+pre-S2 del (4.497)	3. pre-S1+pre-S2 del (nt 3026-3205, 2-55) (0.590)
		4. pre-S1 del (1.986)	4. pre-S1 del (nt 2944-3120) (1.128)
		I. pre-S1 plus pre-S2 del (36.519)	
		II. pre-S1 plus pre-S1+pre-S2 del (6.483)	
		III. pre-S2 plus pre-S1+pre-S2 del (39.030)	
		IV. all three types of pre-S del (41.016)	
75	wild-type	1. **wild-type (97.112)**	1. wild-type (97.112)
		2. pre-S1 del (2.178)	2. pre-S1 del (nt 2854-3021) (0.641)
		3. pre-S2 del (0.679)	3. pre-S2 del (nt 25-54) (0.355)
		4. pre-S1+pre-S2 del (0.031)	4. pre-S1+pre-S2 del (nt 2855-2970, 25-54) (0.007)
		I. pre-S1 plus pre-S2 del (2.857)	
		II. pre-S1 plus pre-S1+pre-S2 del (2.209)	
		III. pre-S2 plus pre-S1+pre-S2 del (0.710)	
		IV. all three types of pre-S del (2.888)	

[a]All the PCR bands visualized in agarose gel were analyzed and listed in descending size order.

[b]The total frequency of pre-S gene DNA in each type of pre-S deletion was shown in descending order (1-4).

[c]The pre-S gene DNA with the highest frequency in each type of pre-S deletion was shown.

[d]The pre-S deletion type above the cut-off percentage (4.643) was shown in bold.

[e]Combination of different types of pre-S deletions was shown (I-IV).

Abbreviations: nt, nucleotide; del, deletion.

Table S2. Univariate and multivariate analyses of pre-S deletion type for overall survival in 75 HBV-related HCC patients

	Univariate Analysis			Multivariate Analysis		
**Characteristics**	**HR**	**95% CI**	**P value**	**HR**	**95% CI**	**P value**
Age (years) (>50 vs. ≤50)	0.764	0.270-2.159	0.6109			
Gender (men vs. women)	1.462	0.191-11.179	0.7143			
Smoking (yes vs. no)	1.004	0.357-2.821	0.9947			
Alcohol (yes vs. no)	2.048	0.760-5.524	0.1566			
HBsAg (positive vs. negative)[a]						
HBeAg (positive vs. negative)[b]	0.635	0.083-4.861	0.6616			
HBV genotype (B vs. C)	1.296	0.292-5.752	0.7332			
HBV DNA (IU/mL) (>1×10^4 vs. ≤1×10^4)[c]	1.682	0.561-5.043	0.3537			
Albumin (g/dL) (>3.8 vs. ≤3.8)	0.425	0.135-1.341	0.1444			
AST (U/L) (>34 vs. ≤34)	1.323	0.298-5.864	0.7126			
ALT (U/L) (>40 vs. ≤40)	0.849	0.307-2.349	0.7530			
AFP (ng/mL) (>400 vs. ≤400)	2.542	0.937-6.895	0.0669			
Tumor size (cm) (>5 vs. ≤5)	1.605	0.581-4.437	0.3615			
Tumor encapsulation (yes vs. no)[d]	0.386	0.124-1.199	0.0997			
Lymph node involvement (yes vs. no)	0.513	0.067-3.899	0.5186			
Portal vein thrombosis (yes vs. no)	1.981	0.444-8.834	0.3701			
Vascular invasion (yes vs. no)	1.930	0.680-5.480	0.2169			
Distant metastasis (yes vs. no)	3.336	0.924-12.040	0.0658			
Steatosis grade (2/3 vs. 0/1)[e]	21.354	1.329-343.018	0.0307*			
Metavir inflammation score (2/3 vs. 0/1)[f]	1.474	0.284-7.645	0.6438			
Ishak fibrosis score (4/5/6 vs. 0/1/2/3)[g]	1.153	0.324-4.100	0.8263			
Child-Pugh cirrhosis score (B/C vs. A)	2.953	1.044-8.356	0.0413*	2.974	1.035-8.549	0.0430*
CLIP score (4/5/6 vs. 0/1/2/3)	5.233	0.675-40.579	0.1133			

Tumor differentiation grade (3/4 vs. 1/2)	0.818	0.291-2.301	0.7040			
BCLC stage (C/D vs. A/B)	1.314	0.296-5.834	0.7199			
AJCC TNM stage (IIIA/IIIB/IIIC/IVA/IVB vs. I/II)	3.113	1.053-9.201	0.0400*	3.146	1.041-9.510	0.0423*
Antiviral therapy after surgery (yes vs. no)	0.746	0.279-1.993	0.5590			
Del spanning pre-S gene segments (yes vs. no)	1.051	0.373-2.962	0.9250			
Only pre-S1 del (yes vs. no)	1.324	0.454-3.868	0.6073			
Only pre-S2 del (yes vs. no)	0.814	0.107-6.206	0.8423			
Only pre-S1+pre-S2 del (yes vs. no)[h]						
Both pre-S1 and pre-S2 del (yes vs. no)	3.216	0.708-14.615	0.1304			
Both pre-S1 and pre-S1+pre-S2 del (yes vs. no)	1.606	0.209-12.326	0.6487			
Both pre-S2 and pre-S1+pre-S2 del (yes vs. no)	0.000	0.000	0.9937			
All three types of pre-S del (yes vs. no)	0.302	0.039-2.366	0.2542			
Del spanning the pre-S1 gene segment (yes vs. no)	1.110	0.402-3.065	0.8407			
Del spanning the pre-S2 gene segment (yes vs. no)	0.818	0.283-2.366	0.7104			

[a]There were no patients negative for HBsAg for analysis.

[b]Only 71 patients with available data were analyzed.

[c]Only 74 patients with available data were analyzed.

[d]Only 62 patients with available data were analyzed.

[e]Only 25 patients with available data were analyzed and thus excluded from multivariate analysis.

[f]Only 44 patients with available data were analyzed.

[g]Only 56 patients with available data were analyzed.

[h]There were no patients with only pre-S1+pre-S2 del for analysis.

*, P value<0.05.

Abbreviations: HR, hazard ratio; CI, confidence interval; del, deletion.

Table S3. Univariate and multivariate analyses of pre-S deletion percentage for overall survival in 75 HBV-related HCC patients

	Univariate Analysis			Multivariate Analysis		
**Characteristics**	**HR**	**95% CI**	**P value**	**HR**	**95% CI**	**P value**
Child-Pugh cirrhosis score (B/C vs. A)	2.953	1.044-8.356	0.0413*	2.974	1.035-8.549	0.0430*
AJCC TNM stage (IIIA/IIIB/IIIC/IVA/IVB vs. I/II)	3.113	1.053-9.201	0.0400*	3.146	1.041-9.510	0.0423*
Pre-S1 del percentage[a]						
II (>1.895) vs. I (≤1.895)[b]	1.630	0.402-6.603	0.4938			
III (>5.629) vs. I (≤1.895)	1.061	0.214-5.263	0.9426			
IV (>28.270) vs. I (≤1.895)	1.396	0.306-6.367	0.6664			
Pre-S2 del percentage						
II (>0.643) vs. I (≤0.643)	1.482	0.387-5.669	0.5658			
III (>1.824) vs. I (≤0.643)	1.276	0.317-5.129	0.7315			
IV (>13.666) vs. I (≤0.643)	1.100	0.241-5.011	0.9024			
Pre-S1+pre-S2 del percentage						
II (>0.015) vs. I (≤0.015)	1.126	0.311-4.070	0.8567			
III (>0.442) vs. I (≤0.015)	0.766	0.171-3.439	0.7282			
IV (>2.975) vs. I (≤0.015)	0.717	0.157-3.287	0.6687			
Pre-S1 plus pre-S2 del percentage						
II (>2.725) vs. I (≤2.725)	1.846	0.473-7.209	0.3776			
III (>18.539) vs. I (≤2.725)	0.295	0.031-2.846	0.2913			
IV (>49.938) vs. I (≤2.725)	1.696	0.399-7.206	0.4745			
Pre-S1 plus pre-S1+pre-S2 del percentage						
II (>2.043) vs. I (≤2.043)	0.728	0.178-2.986	0.6595			
III (>6.332) vs. I (≤2.043)	1.108	0.297-4.136	0.8782			
IV (>44.201) vs. I (≤2.043)	0.767	0.164-3.589	0.7360			
Pre-S2 plus pre-S1+pre-S2 del percentage						

II (>0.689) vs. I (≤0.689)	1.819	0.500-6.615	0.3639
III (>2.109) vs. I (≤0.689)	1.137	0.283-4.563	0.8564
IV (>24.995) vs. I (≤0.689)	0.771	0.139-4.292	0.7668
All three types of pre-S del percentage			
II (>2.828) vs. I (≤2.828)	1.035	0.288-3.718	0.9580
III (>21.666) vs. I (≤2.828)	0.208	0.023-1.866	0.1606
IV (>58.035) vs. I (≤2.828)	1.499	0.391-5.747	0.5548

[a]Patients were divided into quarters of the distribution of indicated type of pre-S del percentage from the lowest to the highest (designated I to IV, respectively).

[b]Threshold percentage of indicated pre-S del type for each quarter was shown in parenthesis.

*, P value<0.05.

Abbreviations: HR, hazard ratio; CI, confidence interval; del, deletion.

Table S4. Univariate and multivariate analyses of combined pre-S deletion for overall survival in 75 HBV-related HCC patients

	Univariate Analysis			Multivariate Analysis		
**Characteristics**	**HR**	**95% CI**	**P value**	**HR**	**95% CI**	**P value**
Child-Pugh cirrhosis score (B/C vs. A)	2.953	1.044-8.356	0.0413*	2.974	1.035-8.549	0.0430*
AJCC TNM stage (IIIA/IIIB/IIIC/IVA/IVB vs. I/II)	3.113	1.053-9.201	0.0400*	3.146	1.041-9.510	0.0423*
Combined pre-S del[a]						
com-II[b] vs. com-I						
com-III vs. com-I	1.101	0.297-4.074	0.8859			
com-IV vs. com-I	0.597	0.132-2.702	0.5029			

[a]Patients were divided into four groups based on pre-S del types and percentages: com-I, del spanning the pre-S2 gene segment (no) & pre-S2 plus pre-S1+pre-S2 del percentage (I/II/III); com-II, del spanning the pre-S2 gene segment (no) & pre-S2 plus pre-S1+pre-S2 del percentage (IV); com-III, del spanning the pre-S2 gene segment (yes) & pre-S2 plus pre-S1+pre-S2 del percentage (I/II/III); and com-IV, del spanning the pre-S2 gene segment (yes) & pre-S2 plus pre-S1+pre-S2 del percentage (IV).

[b]There were no patients falling into the com-II group for analysis.

*, P value<0.05.

Abbreviations: HR, hazard ratio; CI, confidence interval; del, deletion; com, combined.
