# Supplementary figures and images for: Next-Generation Sequencing-Based Quantitative Detection of Hepatitis B Virus Pre-S Mutants in Plasma Predicts Hepatocellular Carcinoma Recurrence
Source: Viruses. 2020 Jul 24;12(8):796. doi: 10.3390/v12080796 (PMC7472021; doi:10.3390/v12080796)

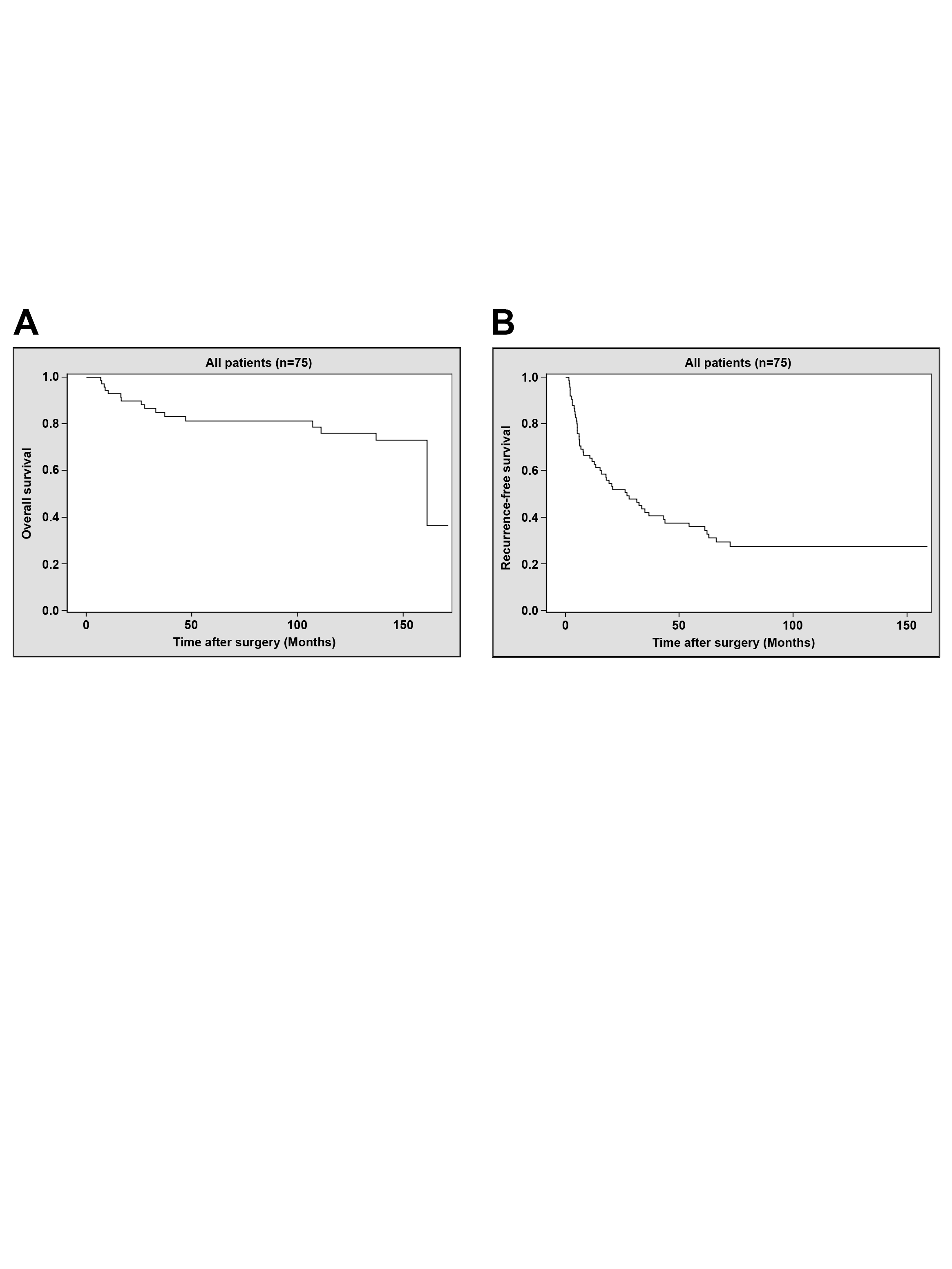

Supplement: Supplementary file 1 [file viruses-12-00796-s001.zip › viruses-850144-for conversion-suppl_/Figure S1.tif]

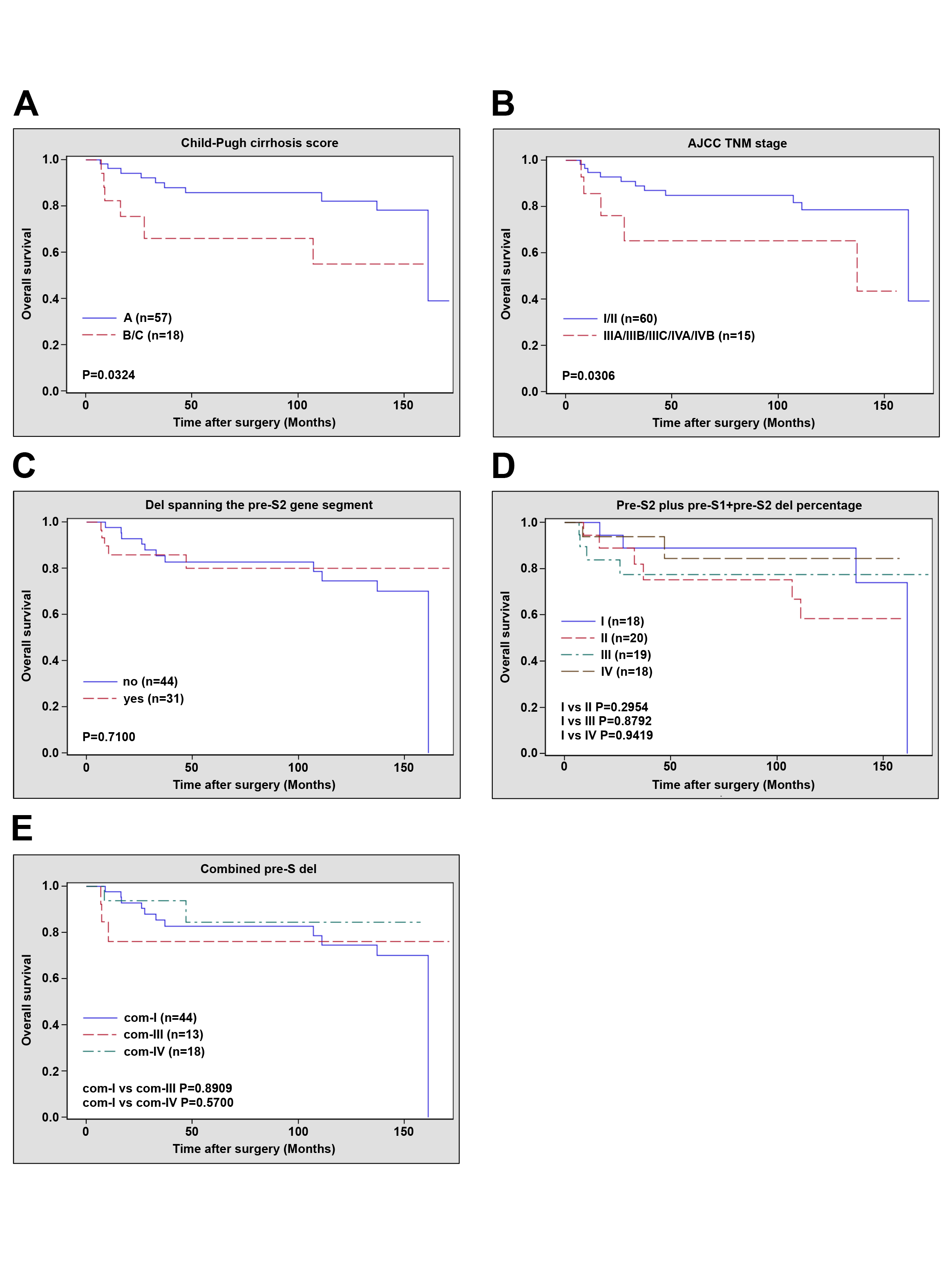

Supplement: Supplementary file 1 [file viruses-12-00796-s001.zip › viruses-850144-for conversion-suppl_/Figure S2.tif]
